# New molecular mechanisms in cholangiocarcinoma: signals triggering interleukin-6 production in tumor cells and KRAS co-opted epigenetic mediators driving metabolic reprogramming

**DOI:** 10.1186/s13046-022-02386-2

**Published:** 2022-05-26

**Authors:** Leticia Colyn, Gloria Alvarez-Sola, M. Ujue Latasa, Iker Uriarte, Jose M. Herranz, Maria Arechederra, George Vlachogiannis, Colin Rae, Antonio Pineda-Lucena, Andrea Casadei-Gardini, Federica Pedica, Luca Aldrighetti, Angeles López-López, Angeles López-Gonzálvez, Coral Barbas, Sergio Ciordia, Sebastiaan M. Van Liempd, Juan M. Falcón-Pérez, Jesus Urman, Bruno Sangro, Silve Vicent, Maria J. Iraburu, Felipe Prosper, Leonard J. Nelson, Jesus M. Banales, Maria Luz Martinez-Chantar, Jose J. G. Marin, Chiara Braconi, Christian Trautwein, Fernando J. Corrales, F. Javier Cubero, Carmen Berasain, Maite G. Fernandez-Barrena, Matias A. Avila

**Affiliations:** 1 Hepatology Program, CIMA, Universidad de Navarra, Pamplona, Spain; 2 CIBERehd, Madrid, Spain; 3 Instituto de Investigaciones Sanitarias de Navarra IdiSNA, Pamplona, Spain; 4 Division of Surgery and Cancer, Imperial College London, London, UK; 5 Institute of Cancer Sciences, The University of Glasgow, Glasgow, UK; 6 Molecular Therapies Program, CIMA, Universidad de Navarra, Pamplona, Spain; 7 Department of Oncology, San Raffaele Hospital, Milan, Italy; 8 Department of Experimental Oncology, Pathology Unit, IRCCS San Raffaele Scientific Institute, Milan, Italy; 9 Hepatobiliary Surgery Division, Vita-Salute San Raffaele University, IRCCS San Raffaele Hospital, Milan, Italy; 10 Centro de Metabolómica y Bioanálisis (CEMBIO), Facultad de Farmacia University San Pablo CEU, Boadilla del Monte, Spain; 11 Functional Proteomics Laboratory, CNB-CSIC, Proteored-ISCIII, Madrid, Spain; 12 Exosomes Laboratory and Metabolomics Platform, CIC bioGUNE-BRTA, Derio, Spain; 13 Ikerbaske, Basque Foundation for Science, Bilbao, Spain; 14 Gastroenterology Department, Hospital Universitario de Navarra, Pamplona, Spain; 15 Hepatology Unit, Clínica Universidad de Navarra, Pamplona, Spain; 16 Solid Tumors Program, CIMA, Universidad de Navarra, Pamplona, Spain; 17 CIBERonc, Madrid, Spain; 18 Department of Biochemistry and Genetics, University of Navarra, Pamplona, Spain; 19 Oncohematology Program, CIMA, Universidad de Navarra, Pamplona, Spain; 20 Institute of Engineering, School of Engineering, Faraday Building, The University of Edimburgh, Edinburgh, Scotland, UK; 21 Department of Liver and Gastrointestinal Diseases, Biodonostia Health Research Institute - Donostia University Hospital, Ikerbasque, San Sebastian, Spain; 22 Liver Research Laboratory, CIC bioGUNE, Derio, Spain; 23 Physiology and Pharmacology Department, HEVEPHARM, IBSAL, University of Salamanca, Salamanca, Spain; 24 Beatson West of Scotland Cancer Center, Glasgow, UK; 25 Department of Internal Medicine III, University Hospital, RWTH Aachen, Aachen, Germany; 26 Department of Immunology, Ophthalmology and ENT, School of Medicine, Complutense University, Madrid, Spain

**Keywords:** Cholangiocarcinoma, Bile, Inflammation, Interleukin-6, KRAS, G9a histone methyl-transferase, Serine-glycine pathway, Metabolic reprogramming

## Abstract

**Background:**

Cholangiocarcinoma (CCA) is still a deadly tumour. Histological and molecular aspects of thioacetamide (TAA)-induced intrahepatic CCA (iCCA) in rats mimic those of human iCCA. Carcinogenic changes and therapeutic vulnerabilities in CCA may be captured by molecular investigations in bile, where we performed bile proteomic and metabolomic analyses that help discovery yet unknown pathways relevant to human iCCA.

**Methods:**

Cholangiocarcinogenesis was induced in rats (TAA) and mice (*Jnk*^*Δhepa*^ + CCl_4_ + DEN model). We performed proteomic and metabolomic analyses in bile from control and CCA-bearing rats. Differential expression was validated in rat and human CCAs. Mechanisms were addressed in human CCA cells, including Huh28-KRAS^G12D^ cells. Cell signaling, growth, gene regulation and [U-^13^C]-D-glucose-serine fluxomics analyses were performed. In vivo studies were performed in the clinically-relevant iCCA mouse model.

**Results:**

Pathways related to inflammation, oxidative stress and glucose metabolism were identified by proteomic analysis. Oxidative stress and high amounts of the oncogenesis-supporting amino acids serine and glycine were discovered by metabolomic studies. Most relevant hits were confirmed in rat and human CCAs (TCGA). Activation of interleukin-6 (IL6) and epidermal growth factor receptor (EGFR) pathways, and key genes in cancer-related glucose metabolic reprogramming, were validated in TAA-CCAs. In TAA-CCAs, G9a, an epigenetic pro-tumorigenic writer, was also increased. We show that EGFR signaling and mutant KRAS^G12D^ can both activate IL6 production in CCA cells. Furthermore, phosphoglycerate dehydrogenase (PHGDH), the rate-limiting enzyme in serine-glycine pathway, was upregulated in human iCCA correlating with *G9a* expression. In a G9a activity-dependent manner, KRAS^G12D^ promoted PHGDH expression, glucose flow towards serine synthesis, and increased CCA cell viability. KRAS^G12D^ CAA cells were more sensitive to PHGDH and G9a inhibition than controls. In mouse iCCA, G9a pharmacological targeting reduced PHGDH expression.

**Conclusions:**

In CCA, we identified new pro-tumorigenic mechanisms: Activation of EGFR signaling or KRAS mutation drives IL6 expression in tumour cells; Glucose metabolism reprogramming in iCCA includes activation of the serine-glycine pathway; Mutant KRAS drives PHGDH expression in a G9a-dependent manner; PHGDH and G9a emerge as therapeutic targets in iCCA.

**Supplementary Information:**

The online version contains supplementary material available at 10.1186/s13046-022-02386-2.

## Background

Cholangiocarcinoma (CCA) is the most common cancer of the biliary tract and the second most frequent hepatic malignancy [1]. Regardless of its anatomical origin, intrahepatic (iCCA), perihilar or distal (extrahepatic CCA, eCCA), this neoplasia is a devastating disease. Its diagnosis is often made at an advanced stage when surgery, the only potentially curative intervention, often cannot be performed whereas systemic therapies are mainly palliative [1, 2]. CCAs are molecularly heterogeneous tumors, and this characteristic extends beyond their anatomical classification. Recent transcriptomic, genomic, proteogenomic and epigenomic analyses have identified different molecular subclasses indicative of potential carcinogenic mechanisms leading to CCA development [3, 4]. The complex molecular profile of CCAs may underlie their high resistance to chemotherapy, targeted agents and immunotherapy. Therefore a better understanding of the cellular and molecular mechanisms of cholangiocarcinogenesis is needed to identify key targets and leverage therapeutic efficacy [5–8].

Diagnosis of CCA varies with anatomical location, and is usually based on clinical and biochemical analyses in combination with radiologic evaluation that requires a pathologic assessment in most patients [1]. The use of liver biopsies is limited since their sensitivity depends on size and location and patients physical status [9]. Several diagnostic tools are used to detect biliary malignancies including imaging techniques as well as endoscopic retrograde cholangiopancreatography (ERCP) [1, 10, 11]. However, in spite of recent improvements in cholangioscopy and biopsy acquisition, early and accurate diagnosis of CCA remains a challenge [2, 9, 11]. In view of this situation great efforts are being made for the identification of reliable blood-borne biomarkers for CCA detection [12–14]. Some commonly used diagnostic procedures such as ERCP also enable bile collection. In principle, molecules released into bile from tumor cells within the biliary tract would be more concentrated than in blood. For instance, bile cell-free DNA (cfDNA) may include DNA from pre-malignant or malignant cells located along in the biliary tract, and its analysis can have robust diagnostic potential as recently demonstrated by us and others [15–17]. Similarly, bile metabolomic and proteomic studies may also detect tumour-related or tumour-elicited molecules with diagnostic implications [18–20]. However, molecular analyses of bile may not only be potentially useful for CCA detection, but also for the identification of carcinogenic mechanisms, therapeutic vulnerabilities and a better understanding of CCA biology. Under these premises, we have performed a comprehensive proteomic analysis of bile in the thioacetamide (TAA) rat model of cholangiocarcinogenesis, which mimics the multi-step development of human iCCA in a context of chronic liver damage, inflammation and desmoplastic reaction, reproducing the histological progression of human CCA from biliary dysplasia to carcinoma. Importantly, the rat TAA model of CCA also captures key molecular alterations observed in human CCA [21]. These analyses led us to uncover novel mechanisms behind the production of key tumorigenic inflammatory mediators for CCA such as interleukin 6 (IL6) [22, 23], or mediating pro-carcinogenic metabolic reprogramming [24], involving the activation of KRAS-mitogen activated protein kinase (MAPK) signaling, a central event in cholangiocarcinogenesis [25, 26]. We also identified new KRAS tumorigenic pathways in CCA cells implicating epigenetic mechanisms, and confirmed the therapeutic potential of their pharmacological targeting.

## Methods

### Thioacetamide (TAA) model of cholangiocarcinogenesis in rats

**Fig. 1:**
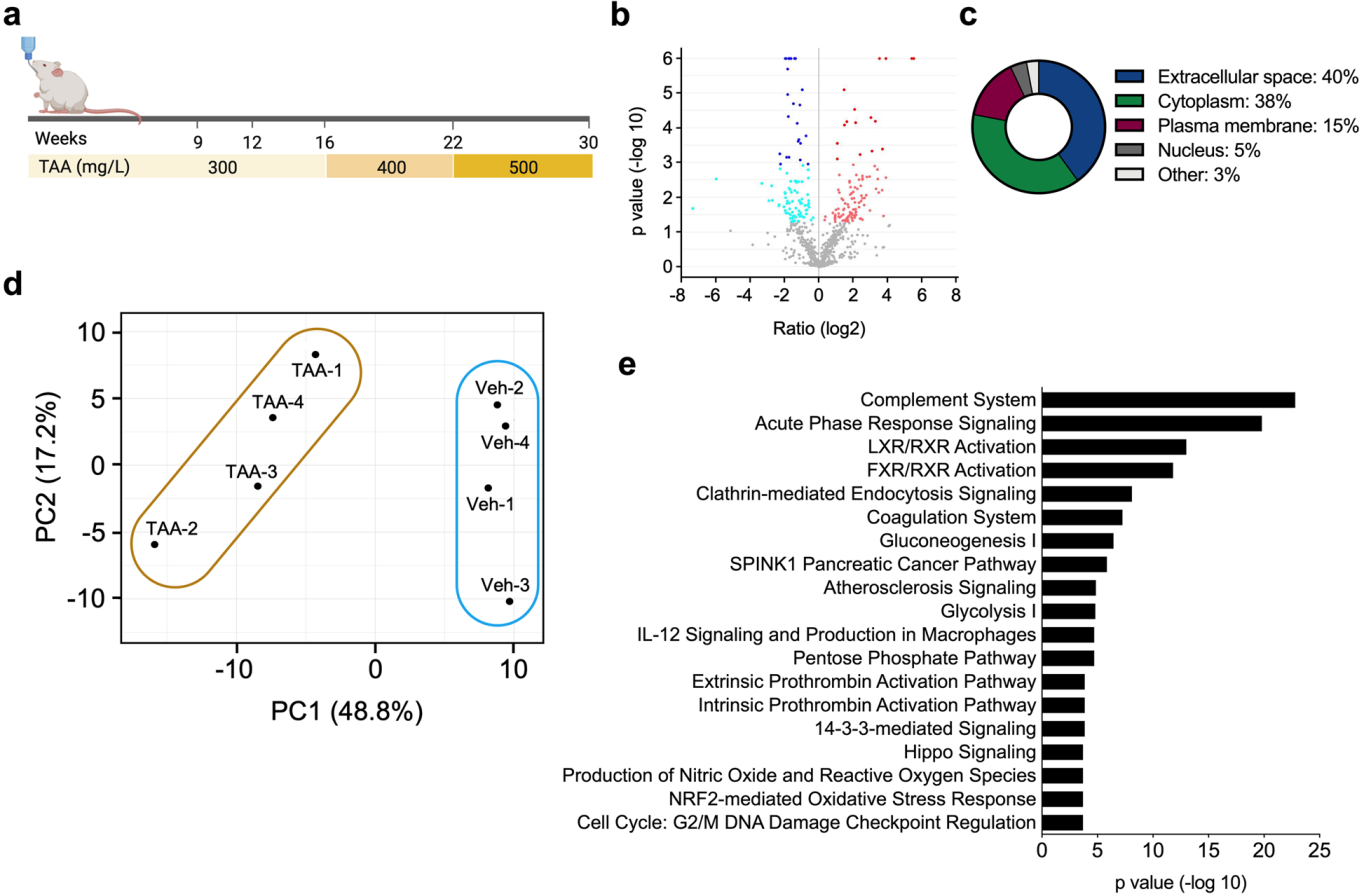
Proteomic analysis of bile in the rat TAA model of CCA development. **a** Schematic representation of the rat TAA model of CAA implemented in this study. **c** Pie chart showing the classification of proteins identified as differentially represented in bile from control and CCA bearing rats (30 weeks) according to their cellular localization. **b** Volcano plot (−log10 [*p*-value] and log2 [fold-change]) of the proteins found in bile from rats with CCA compared with control animals. **d** Principal component analysis (PCA) of bile proteomic data from control (Veh-1-4) and TTA (TAA-1-4) treated rats. **e** Ingenuity pathway analysis (IPA) of the differentially represented proteins between control and CCA bile samples identifying the top enriched categories of canonical pathways. Created with BioRender.com

Eight-week-old male Sprague-Dawley rats (~ 250 g) (Envigo, Barcelona, Spain) were used. Rats were housed in a 12:12 hours light-dark cycle at an ambient temperature of 22 °C with food and water available ad libitum. TAA administration was as previously reported with some adaptations [21]. Briefly, TAA (Sigma, St. Louis MO, USA) was administered in sweetened drinking water (sucrose solution 50 g/L) for 30 weeks in a concentration range escalating from 300 to 500 mg/L (*n* = 6). Control animals (*n* = 4) were given sweetened drinking water. The dose of TAA was progressively increased from 300 to 500 mg/L depending on the weight loss associated with administration of TAA as shown in Fig. 1a. At the end of the treatment rats were anesthetized, abdominal laparotomy was performed and the common bile duct was cannulated for bile collection as previously described [27]. Blood was drawn from the retro orbital plexus, and then animals were sacrificed by cervical dislocation. Liver samples were collected, and snap frozen in liquid nitrogen or formalin-fixed and paraffin embedded (FFPE) for histological analyses.

### Mouse CCA model and treatments

The *Jnk*^Δhepa^ mice, in a C57BL/6 J background, were generated as previously described [28, 29]. At 14 days of age mice received 25 mg/kg (i.p.) of diethyl-nitrosamine (DEN) and from week 8 until week 22 were treated with CCl_4_ (0.5 mL/kg, i.p.) twice per week. From week 18 until week 22 one group of mice (*n* = 6) were treated with CM272 (5 mg/kg, i.p.) daily, control mice received the same volume of PBS, as described [30]. All mice were sibling littermates. Animals were housed and fed a chow diet (Envigo, Valencia, Spain) under standard conditions.

### Tissue staining and immunohistochemistry

Sections obtained from FFPE rat liver tissues were used for H&E and Picro-Sirius Red staining as previously reported [31]. Mouse liver tissues were obtained and processed for immunohistochemical analyses as we described [30]. Human CCA tissue samples were obtained from patients with iCCA (*n* = 41) that underwent surgical resection at San Raffaele Hospital, Milan, Italy. Immunostainings were performed as we described [30, 31] using the antibodies listed in Supplementary Table 1. Quantitative evaluation was performed essentially as previously described using histological criteria in a blinded manner [31]. Intensity of the signal was scored as: 0 = no expression; 1 = weak expression; 2 = intermediate expression; 3 = high expression, and scores according to percentage of cells stained were: 0 (0–9% cells staining), 1 (10–40%), 2 (41–60%) and 3 (61–100%). The final score was obtained by the multiplication of the intensity score by the percentage score. Tumor grade was established upon anatomopathological examination of tissue sections according to the WHO Classification of Tumours, Digestive System Tumours, 5th edition.

### Bile proteomic analyses

Bile samples (30 μl, *n* = 4 control and 4 TAA-treated rats) were processed and prepared for analysis as we described [32]. Proteomic analyses and data processing were performed essentially as we previously reported [20, 33]. Functional analyses were performed with Ingenuity Pathway Analysis, IPA (Quiagen, Hilden, Germany).

### Cell lines and in vitro studies

The characteristics and origin of the CCA cell lines used in this study have been described before [34]. Cells were grown as reported [30]. Huh28 cells expressing mutant KRAS (KRAS^G12D^), and control Huh28 cells (Lac-Z), were generated by infection with lentiviral vectors from OriGene Technologies (Rockville, MD, USA) produced by Genscript Biotech (Piscataway, NJ, USA) as we previously described [35]. For serine deprivation experiments cells were cultured as described [36]. Calculation of growth inhibitory 50 (GI_50_) concentrations of NCT-503 (Sigma), synergistic growth inhibitory effects between CM-272 and NCT-503, cell proliferation studies, soft agar (anchorage-independent) cell growth experiments and colony formation analyses were performed as we previously reported [30, 31]. For quantification of colony formation analyses at the end of treatments plates were washed once with PBS, fixed with 3.7% formaldehyde and stained with Crystal violet. Stained wells were de-stained with 10% acetic acid and absorbances were read in a spectrophotometer at 570 nm wavelength. For IL6 gene expression and IL6 protein analyses CCA cells were treated with HB-EGF (#E4643, Sigma). IL6 concentrations in cells’ conditioned media were analyzed with the BD OptEIA™ human IL6 ELISA set from BD Biosciences (#555220, Franklin Lakes, NJ, USA). Where indicated, MutKRAS cells were treated with the phosphatidyl kinase 3 (PI3K) inhibitor LY294002 (20 μM) or the mitogen-activated protein kinase kinase (MEK) inhibitor PD98059 (20 μM) (both from Calbiochem, San Diego, CA, USA) for 6 h. *PHGDH* gene expression was knocked-down in CCA cells by transfection with specific siRNAs (siPHGDH) (Sigma) as previously described [30]. Subcellular fractionation analyses were performed using the NE-PERTM nuclear and Cytoplasmic Extraction Reagents (#78833) from ThermoFisher (Waltham, MA, USA) as we described [31]. The patient’s derived organoids obtained from an advanced iCCA used in this study have been described before [37]. Organoids (30 μL of growth factor reduced matrigel containing 6000 cells) were seeded in 96-well cell culture plates; after matrigel solidified it was overlaid with 70 μL of complete human organoid medium. Complete medium was refreshed once after 24 h. Treatments with CM-272 [31] was added 3 days later and compound-containing medium was further refreshed every 2 days. After 11 days medium was removed and replaced with 100 μL of complete human organoid medium containing 10% CellTiter-Blue Cell Viability Assay (Promega).

### Flux analysis

For glucose flux analyses control and MutKRAS Huh28 cells (7 × 10^5^ cells/plate) were cultured in regular medium (DMEM, 10% FBS plus glutamine and antibiotics) with or without CM-272 (100 nM) for 66 h. Then cells were washed with PBS and transferred to DMEM without glucose (#11966–025, Gibco-ThermoFisher) supplemented with 10% dialyzed FBS (#A338200, Gibco-ThermoFisher) and 4.5 g/L [U-^13^C] D-glucose (#389374, Sigma) for 7 h before metabolite extraction. Sample processing and ultra-performance liquid chromatography (UPLC)-time-of-flight mass spectrometry (ToF-MS) analysis are described in Additional file 1.

### Image analysis

Colonies grown in soft-agar were quantified by image analysis. Images (*n* = 5 per condition) were captured at 10X magnification (Leica microscope, Wetzlar, Germany). Area was calculated by image analysis tools in Fiji/ImageJ software (http://fiji.sc/).

### Statistical analyses

Statistical analysis was performed using GraphPad Prism-v5 software. For comparison between two groups, two-sided unpaired Student’s t-test or Mann–Whitney U-test were used according to sample distribution. All reported *P* values were two-tailed and differences were considered significant when *P* < 0.05.

Additional methodological information is provided as supplementary material.

## Results

### TAA rat model and bile analyses

**Fig. 2:**
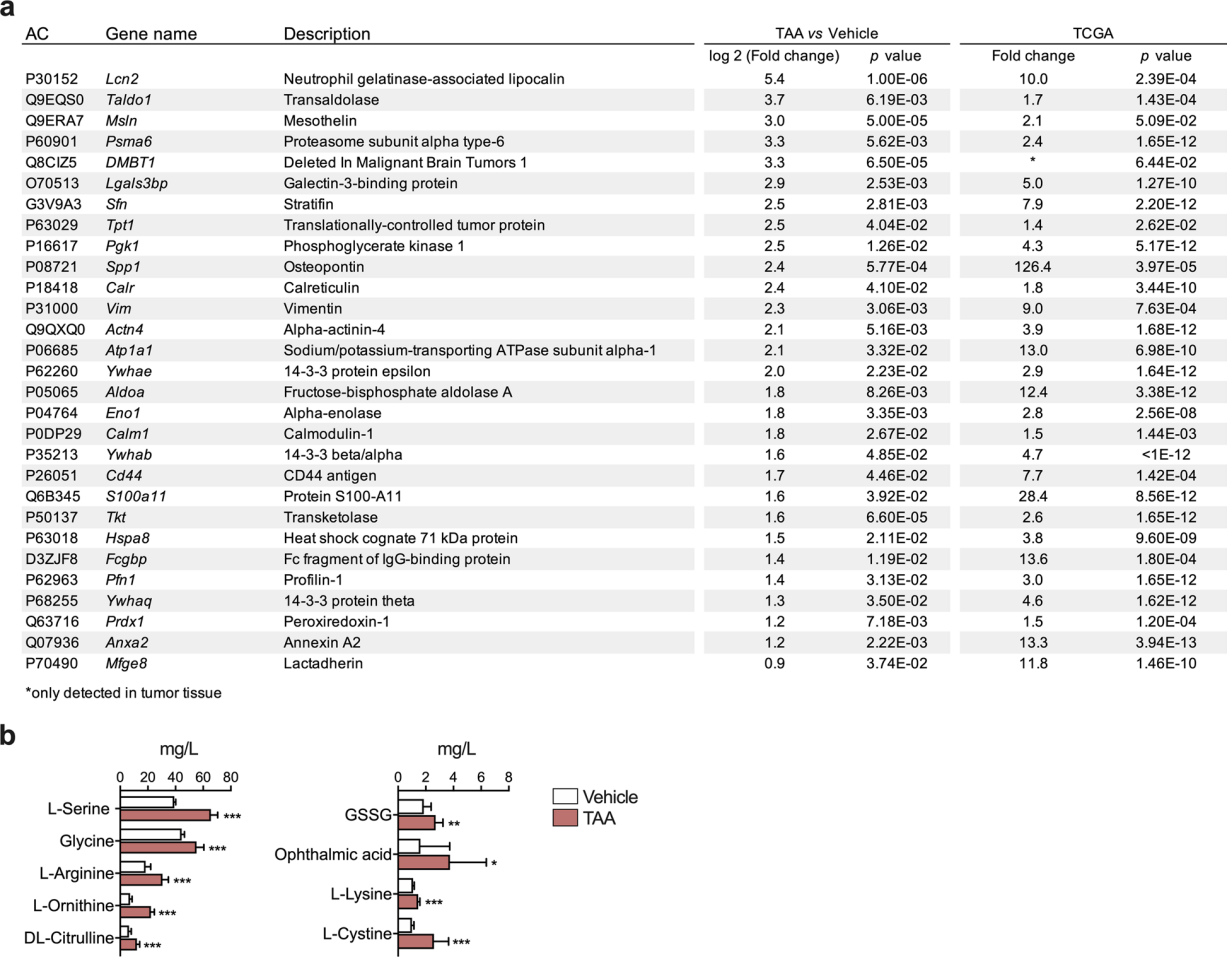
Most relevant proteins and metabolites differentially represented in bile from control and CCA bearing rats. **a** Identity of proteins showing significantly increased concentrations (Fold change > 0.9) in bile from TAA-treated rats vs controls (Vehicle). The expression of the corresponding genes as reported in the TCGA database is shown. AC: accession number. **b** Metabolites showing significantly altered concentrations in bile from TAA-treated rats vs controls (Vehicle). *p < 0.05, **p < 0.01, ***p < 0.001

Rats were administered TAA in drinking water up to 30 weeks (Fig. 1a). Increased liver index, accompanied by hyperbilirubinemia and hypercholanemia was observed as TAA treatment progressed (Supplementary Fig. 1a and b). At 30 weeks, and consistent with the literature [21, 38], all animals developed visible tumors, liver fibrosis and multifocal cytokeratin 19 (CK19)-positive mass forming lesions with a strong desmoplastic reaction (Supplementary Fig. 1c and d). At this end-point bile was collected from control and TAA-treated rats and proteomic analyses were performed (Supplementary Fig. 1e). We identified 212 proteins differentially expressed, of which 111 were upregulated and 101 downregulated in CCA-harboring rats vs controls (Fig. 1b). Most of these proteins corresponded to extracellular or cytoplasmic locations (Fig. 1c). Unsupervised principal component analysis (PCA) of these data clearly discriminated the two groups of samples (Fig. 1d). Ingenuity pathway analysis (IPA) of the differentially represented proteins allowed their preferential classification in certain biological processes, overlapping to a great extent with previous proteomic studies, including ours, on human bile from patients with benign and malignant (CCA) biliary stenoses [33]. The canonical pathways enriched in our IPA analysis identified categories such as inflammation (complement, acute phase response, coagulation), metabolic regulation by nuclear sterols and bile acids receptors, glucose metabolism, oxidative stress, cell signaling and cell cycle/DNA damage response (Fig. 1e). Proteins identified in bile from CCA-harboring rats with a fold-change above 0.9 are shown in Fig. 2a. Based on data from TCGA (https://www.cancer.gov/tcga), the expression of all the corresponding genes (*n* = 29) is significantly upregulated in human CCA (Fig. 2a). Gene expression changes in a broad selection of these proteins were validated by RT-qPCR, and for some of them also by immunohistochemistry, in healthy, peritumoral and tumoral rat CCA tissues (Supplementary Fig. 2a and b). Marked upregulation was observed in CCA tissues for carcinogenic genes, including lipocalin 2 (*LCN2*), mesothelin (*MSLN*), deleted in malignant brain tumors 1 (*DMBT1*), Mac-2-binding protein or L-galectin-3-binding protein (*LGALS3BP*), secreted phosphoprotein 1 or osteopontin (*SPP1*), vimentin (*VIM*), cluster of differentiation 44 (*CD44*), S100 calcium binding protein A1 (*S100A1*), annexin A2 (*ANXA2*), Fc gamma binding protein (*FCGBP*), and members of the 14–3-3 family of intracellular signaling proteins such as 14–3-3σ or stratifin (*SFN*, *YWHAS*) and 14–3-3θ (*YWHAQ*). The expression of other genes frequently upregulated in human iCCA, such as *Sox9*, *Klf5*, *Ctgf* and *Jag1* among others, was also significantly upregulated in TAA-induced CCA tissues (Supplementary Fig. 3).

To further characterize the molecular alterations of the CCA microenvironment that can have a reflection detectable in bile we performed an untargeted CE-MS-based metabolite screening. Interestingly, in CCA-associated bile we observed increased levels of the aminoacids L-serine, glycine, L-arginine, L-ornithine, DL-citrulline, L-lysine and L-cystine (Fig. 2b). On the other hand, in spite of the increased levels of the glutathione precursors L-serine, glycine and L-cystine, we found elevated concentrations of ophthalmic acid, a biomarker of glutathione depletion [39], and of oxidized glutathione (GSSG) (Fig. 2b).

### Identification of new mechanisms involving inflammatory and growth factor crosstalks in CCA

**Fig. 3:**
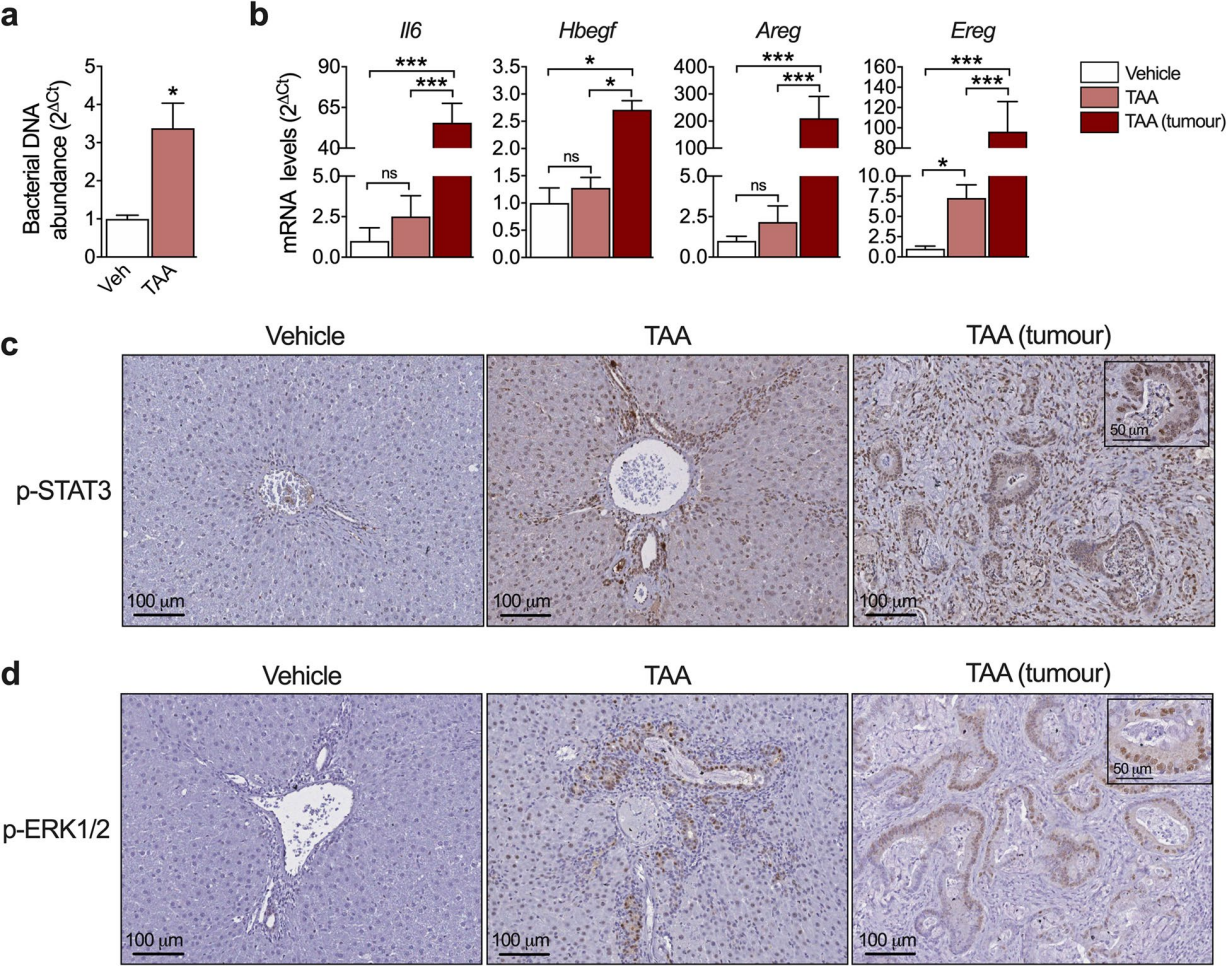
Inflammatory and growth factor-related signaling activation in the TAA rat model of CCA. **a** Quantification of bacterial DNA levels in the livers of control (Veh) and TAA treated rats. *p < 0.05. **b** mRNA levels of *Il6* and the EGFR ligands heparin-binding EGF (*Hbegf*), amphiregulin (*Areg*) and epiregulin (*Ereg*) in liver tissue samples from control rats (Vehicle), peritumour liver tissues and tumour tissues. *p < 0.05, **p < 0.01, ***p < 0.001. **d** Immunohistochemical analysis of p-ERK1/2 in liver tissue samples from control rats (Vehicle), peritumour liver tissues and tumour tissues. Representative images are shown. **c** Immunohistochemical analysis of p-STAT3 in liver tissue samples from control rats (Vehicle), peritumour liver tissues and tumour tissues. Representative images are shown

**Fig. 4:**
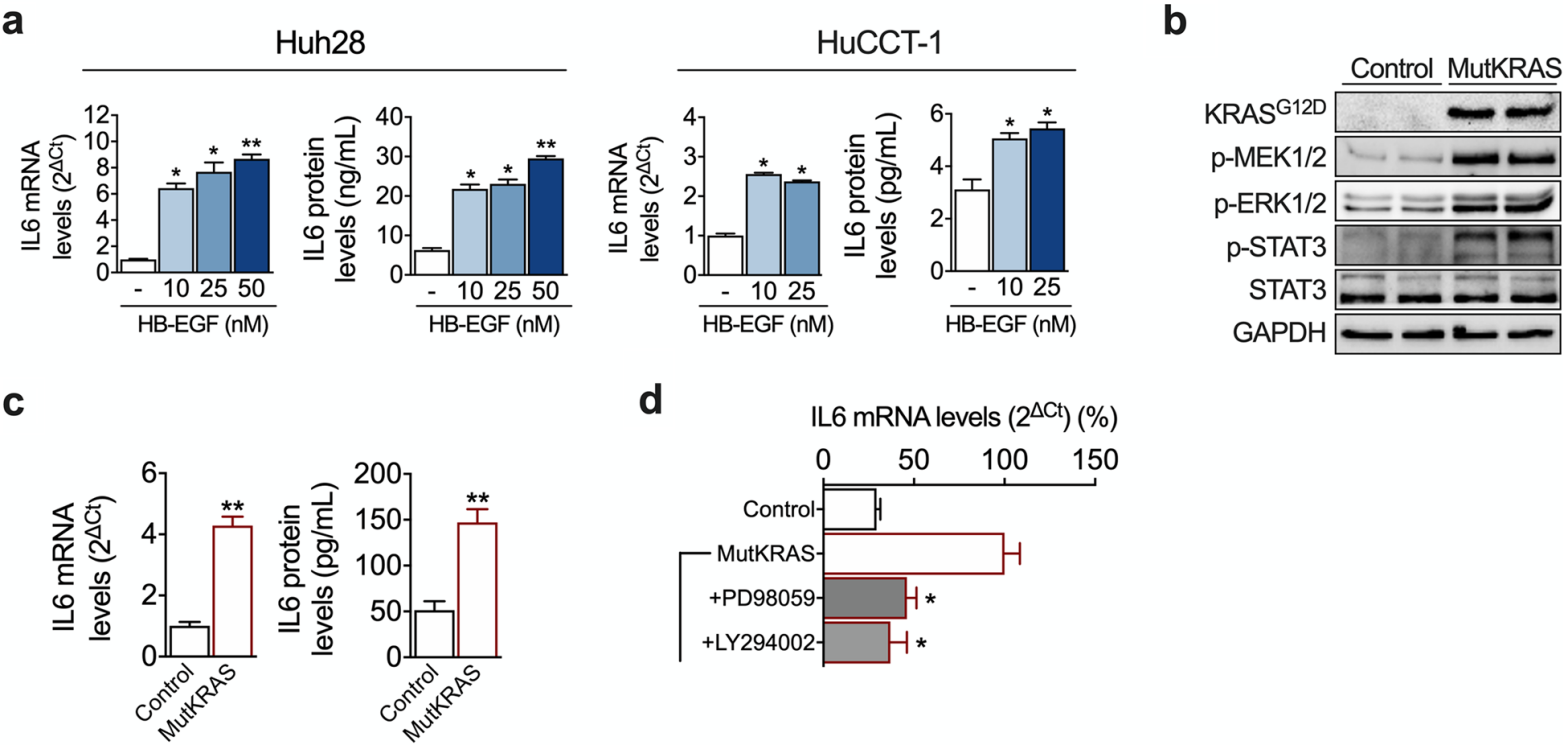
EGFR-KRAS signaling triggers IL6 expression in CCA cells. **a** Effect of HB-EGF on IL6 mRNA expression (12 h treatment) and IL6 protein release (24 h treatment) in HuCCT-1 and Huh28 cells. *p < 0.05, **p < 0.01. **b** Characterization of control and KRAS^G**1**2D^ (MutKRAS) Huh28 cells. Images show representative western blot analyses of KRAS^G12D^, p-MEK1/2, p-ERK1/2, p-STAT3, STAT3 levels, as well as GAPDH levels, as loading control, in both cell lines. **c** Expression levels of IL6 mRNA and IL6 protein concentrations in the conditioned media (24 h culture) of control and MutKRAS Huh28 cells. **p < 0.01. **d** Expression levels of IL6 mRNA in MutKRAS cells treated with PI3K (LY294002) or MEK (PD98059) inhibitors for 6 h. *p < 0.05

Our bile proteomic analysis consistently captured the inflammatory milieu in which CCA develops in the TAA model. Complement activation and acute phase response signaling were among the top categories in the IPA analysis. Recent studies have described the presence and pro-tumorigenic role of bacteria and bacterial products in human CCA tissues [4, 40]. This driving factor was also present in our model, as we were able to detect significantly increased bacterial DNA levels in liver tissues from TAA-treated rats (Fig. 3a). Interleukin 6 (IL6) is a key inflammatory mediator driving cholangiocarcinogenesis [22, 41] to a great extent through the activation of the signal transducer and activator or transcription-3 (STAT3) pathway in CCA tissues [25]. We detected a remarkable upregulation of *Il6* gene expression in rat tumors (Fig. 3b), and a robust staining for phosphorylated STAT3 (p-STAT3) in the nuclei of tumoral cells (Fig. 3c). Within the CCA microenvironment IL6 can be produced by Kupffer cells, tumor-associated macrophages, cancer-associated fibroblasts (CAFs) and also by tumor cells [23, 25]. Noteworthy, previous works in other tumor types identified the RAS pathway as an essential driver of IL6 expression [42, 43]. Besides the frequent oncogenic mutations in *KRAS* found in CCA, the RAS-MAPK pathway can be triggered by additional mechanisms such as the epidermal growth factor receptor (EGFR) signaling system, which is also activated and contributes to cholangiocarcinogenesis [22, 25]. Under these premises, we observed a strong nuclear staining for p-ERK1/2, a read-out of the RAS-MAPK pathway, in rat iCCA tissues (Fig. 3d). Moreover, the expression of the EGFR ligands heparin-binding EGF (*Hbegf*), amphiregulin (*Areg*) and epiregulin (*Ereg*) was also markedly elevated in tumor tissues (Fig. 3b). These observations led us to directly assess the effect of EGFR activation on IL6 expression in human CCA cells. We found that treatment of Huh28 and HuCCT-1 human iCCA cell lines with recombinant HB-EGF resulted in a dose-dependent expression and secretion of IL6 (Fig. 4a). Next, to directly evaluate the role of oncogenic *KRAS* in IL6 expression by CCA cells we developed a KRAS^G12D^ expressing CCA cell line using human Huh28 cells (MutKRAS), which harbor wild type *KRAS* alleles [44]. As expected, MutKRAS cells displayed increased levels of p-MEK and p-ERK1/2, and most interestingly also of p-STAT3 (Fig. 4b). Importantly, MutKRAS expression resulted in increased *IL6* up-regulation and subsequent enhanced release of IL6 to the culture medium (Fig. 4c). To gain further insight into the mechanisms downstream of KRAS involved in IL6 up-regulation we treated MutKRAS cells with inhibitors of PI3K, MEK, p38 MAPK and JNK and evaluated IL6 mRNA levels. As shown in Fig. 4d, inhibition of PI3K or MEK resulted in reduced basal levels of IL6 mRNA, suggesting that different pathways could be involved in IL6 expression in *KRAS* mutated cells. Together, these observations identify the RAS-MAPK pathway, and *KRAS* oncogenic mutation, as drivers for IL6 production in CCA cells.

### Oncogenic metabolic reprogramming in CCA

**Fig. 5:**
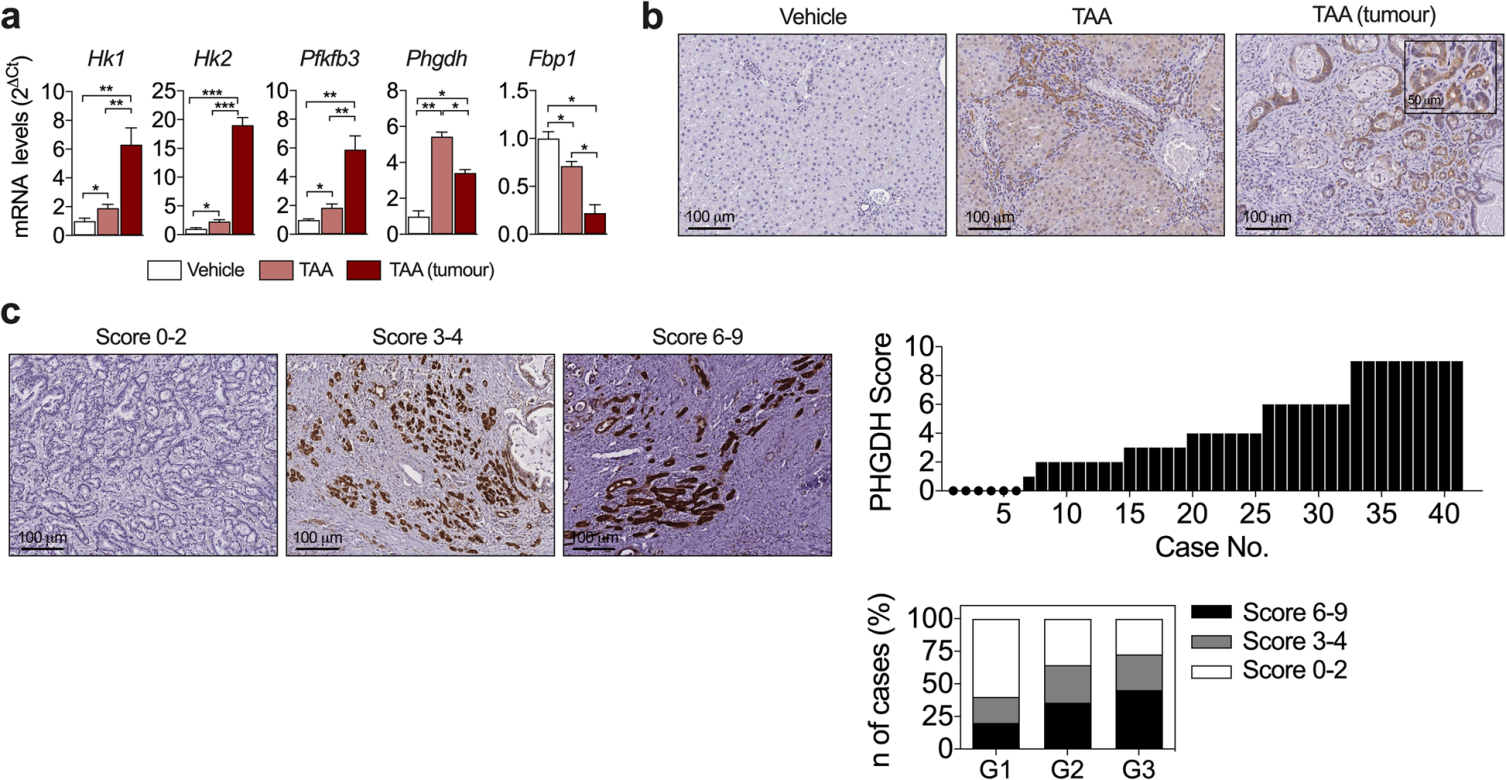
Metabolic reprogramming in experimental CAA (rat TAA model) and human iCCA. **a** Expression of glucose metabolism-related genes in liver tissue samples from control rats (Vehicle), peritumour liver tissues and tumour tissues. *p < 0.05, **p < 0.01, ***p < 0.001. **b** Immunohistochemical analysis of PHGDH in liver tissue samples from control rats (Vehicle), peritumoural liver tissues and tumoural tissues. Representative images are shown. **c** Immunohistochemical analysis of PHGDH in human iCCA tissue samples. Representative images of tumours with 9, 6–4 and 0 PHGDH immunostaining scores are shown. Graphs show the distribution of PHGDH scores among all iCCA tissue samples and according to tumor grade (G1-G3)

Another insight from our proteomic study was the identification by IPA of several categories related to glucose metabolism (Fig. 1e). Metabolic rewiring is a hallmark of cancer cells, and glucose metabolic reprogramming is increasingly recognized to occur in CCA [45]. We observed the upregulation of glycolytic genes such as hexokinase-1 and -2 (*Hk1* and *Hk2*), 6-phosphofructo-2-kinase/fructose-2,6-bisphosphatase 3 (*Pfkfb3*) (Fig. 5a) and aldolase-A (*AldoA*) (Supplementary Fig. 2a), along with the downregulation of the gluconeogenesis rate-limiting gene fructose-1,6-bisphosphatase-1 (*Fbp1*), in chronically-injured liver tissues and in tumours (Fig. 5a). As shown in Fig. 2b, we detected increased concentrations of L-serine and glycine in bile from tumour bearing rats, ascribable to the activity of the serine-glycine synthesis pathway, a glycolytic side-branch frequently triggered in cancer cells in which phosphoglycerate dehydrogenase (*Phgdh*) catalyzes the first and rate-limiting step [24]. We observed increased mRNA levels of *Phgdh* in chronically-injured liver and CCA tissues (Fig. 5a), and PHGDH protein was predominantly detected in both fibrogenic cells in non-tumoural tissues and in the tumour cellular compartment (Fig. 5b). In view of these findings we evaluated PHGDH expression in human CCA. Immunohistochemical analyses revealed that PHGDH was readily detected in most iCCA samples, with very few cases of zero score (Fig. 5c). Interestingly, cases with higher scores for PHGDH expression were more represented among higher grade tumors (Fig. 5c ). From a functional perspective, we observed that when *PHGDH* expression was knocked-down by transfection with specific siRNAs cell growth was reduced (Supplementary Fig. 4).

**Fig. 6:**
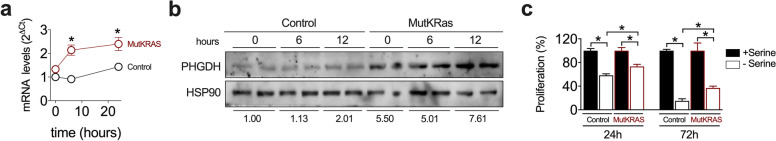
Expression of PHGDH in wild type (control) and KRAS^G12D^ expressing (MutKRAS) Huh28 cells, response to L-serine availability. **a** PHGDH mRNA levels in control and MutKRAS Huh28 cells grown in complete medium (t = 0) and at the indicated time-points after L-serine depletion. *p < 0.05 vs control. **b** PHGDH protein levels were analyzed by western blotting in same samples described in A. Representative blots, including HSP90 analysis as loading control, are shown. **c** Growth of control and MutKRAS Huh28 cells in L-serine depleted medium referenced to cell growth in complete medium. *p < 0.05, **p < 0.01

In cancer cells *PHGDH* upregulation could be driven by *KRAS* activating mutations, thus we tested whether such mechanism would also take place in CCA cells. To this end, we examined the expression of PHGDH in our control and MutKRAS Huh28 CCA cells. We found that *PHGDH* expression was indeed higher in MutKRAS cells, and that this difference became exacerbated when serine was removed from the culture medium (Fig. 6a and b). This response was accompanied by an enhanced capacity of MutKRAS cells to grow in serine-depleted medium compared to control cells (Fig. 6c). Taken together, these findings further validated the occurrence of glucose metabolic reprogramming in CCA and identified the presence of KRAS mutations as candidate drivers for the activation of the serine-glycine pathway in this type of tumour.

### Mechanisms and targeting of KRAS^G12D^-mediated PHGDH expression and growth of CCA cells

**Fig. 7:**
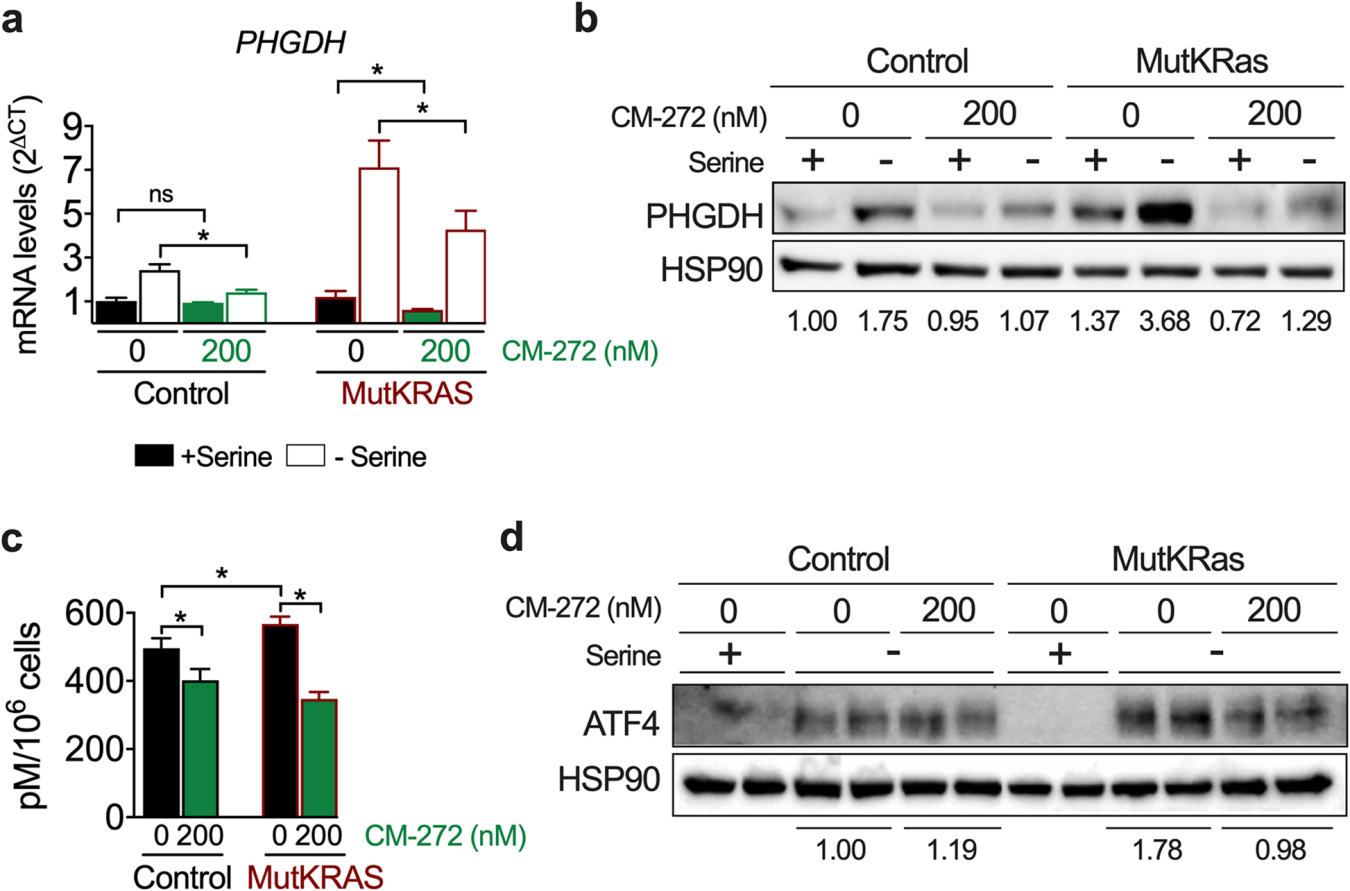
G9a inhibition blunts the adaptive upregulation of *PHGDH* expression to L-serine availability in CCA cells. **a** Control and KRAS^G12D^ expressing (MutKRAS) Huh28 cells were grown in complete medium for 60 h with or without CM-272 (200 nM) and then maintained in complete medium or without L-serine for another 20 h. At this point *PHGDH* mRNA expression was analyzed. *p < 0.05. **b** PHGDH protein levels were analyzed by western blotting in same samples described in a. Representative blots, including HSP90 analysis as loading control, are shown. **c** UPLC-ToF-MS analysis of [U-^**13**^C] glucose flux into serine in control and MutKRAS Huh28 cells treated of not with CM-272 ( 200 nM, 66 h). *p < 0.05. **d** ATF4 protein levels were analyzed by western blotting in same samples described in a. Representative blots, including HSP90 analysis as loading control, are shown

We recently showed that pharmacological targeting of the histone methyltransferase (HMT) G9a with CM-272 downregulated the basal expression levels of *PHGDH* in different CCA cell lines [30]. We confirmed the potent growth inhibitory effects of CM-272 in a tumor organoid derived from the liver biopsy of a chemoresistant iCCA patient [37] (Supplementary Fig. 5). Moreover, we also observed a positive correlation between *G9a* and *PHGDH* gene expression in human iCCA tissues (Supplementary Fig. 6). Therefore, we tested whether KRAS^G12D^-driven *PHGDH* expression could also involve G9a activity. For this, control and MutKRAS Huh28 cells were pre-treated or not with CM-272 and then incubated in serine-depleted medium. As shown in Fig. 7a and b, CM-272 markedly reduced *KRAS*^*G12D*^-driven basal PHGDH expression and blunted its upregulation upon serine reduction in the context of KRAS mutation. We also analyzed the activity of the serine biosynthetic pathway by [U-^13^C] glucose flux analysis using UPLC-ToF-MS. We observed that under basal conditions MutKRAS Huh28 showed increased incorporation of [U-^13^C] glucose into serine compared to control Huh28 cells, and that treatment with CM-272 decreased glucose flux into serine in both cell lines (Fig. 7c). KRAS-driven metabolic rewiring may implicate different pathways and mechanisms such as the regulation of PHGDH by the activating transcription factor 4 (ATF4) [46, 47]. We found that the robust induction of ATF4 protein levels observed in MutKRAS Huh28 cells during serine depletion was attenuated by CM-272 (Fig. 7d). Together, these findings indicate that G9a participates in KRAS-mediated metabolic rewiring in CCA cells.

**Fig. 8:**
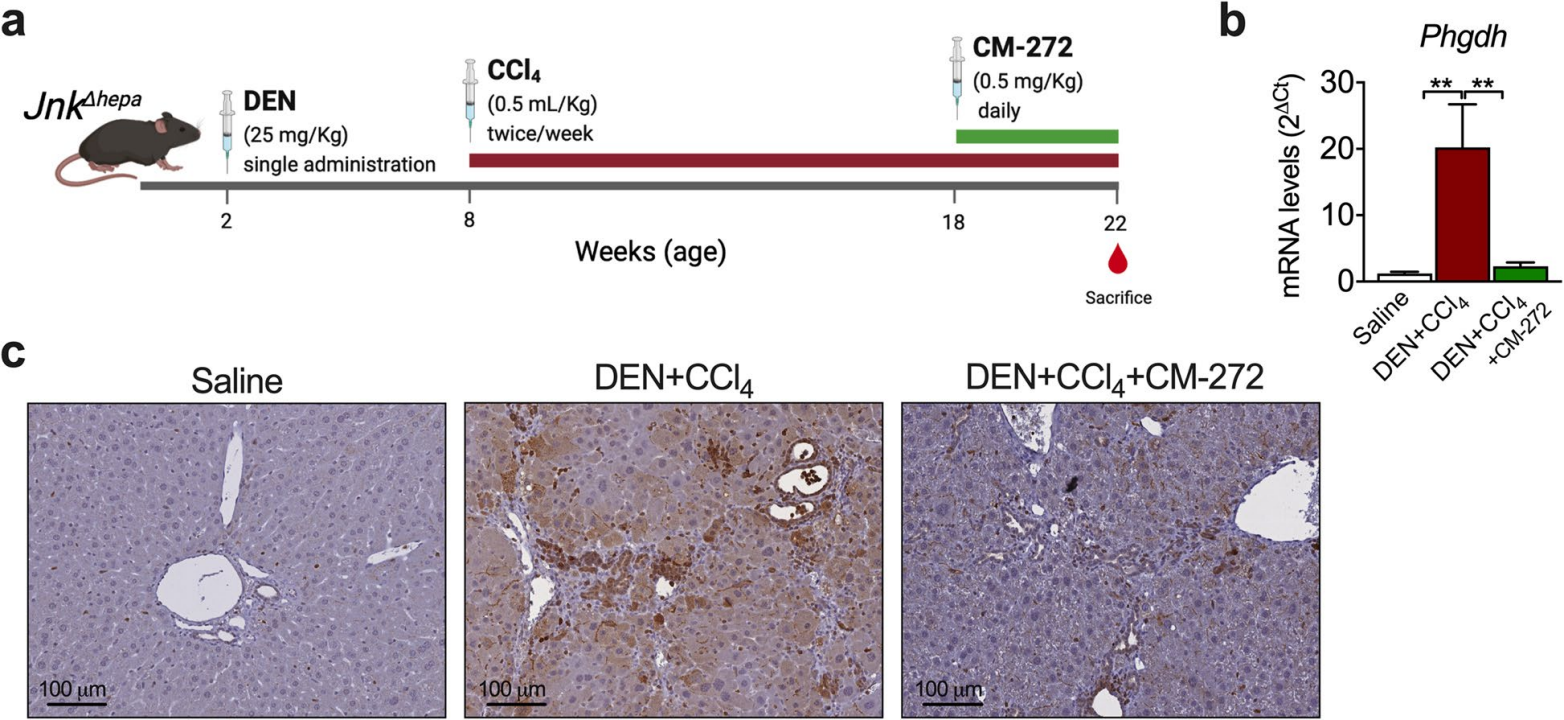
G9a inhibition reduces PHGDH expression in a mouse model of CCA. **a** Diagram showing the experimental model and the treatments applied (*n* = 6 mice per group). **b** *Phgdh* mRNA levels in the liver of wild type mice, *Jnk*^***Δ****hepa*^ mice, *Jnk*^***Δ****hepa*^ mice treated with CCl_4_ and diethylnitrosamine (DEN) (*Jnk*^***Δ****hepa*^ + CCl_4_ + DEN mice) and *Jnk*^***Δ****hepa*^ + CCl_4_ + DEN mice treated with CM-272 as indicated. **p < 0.01. **c** Immunohistochemical detection of PHGDH in liver tissue sections from mice treated as described in **b**. Representative images are shown. Created with BioRender.com

We recently described and validated a model in which mice with hepatocellular c-Jun N-terminal kinase 1/2 (*Jnk1/2*) deletion (*Jnk*^*Δhepa*^) treated with CCl_4_ and DEN (*Jnk*^*Δhepa*^ + CCl_4_ + DEN model) develop transplantable CCAs in a context of inflammation, fibrosis and robust MAPK activation in tumoral tissues [28, 48] (Fig. 8a). We also demonstrated high G9a expression in CCA cells and a potent antitumoural effect of CM-272 in this model [30]. Now, we examined hepatic *Phgdh* expression in wild type, *Jnk*^*Δhepa*^, *Jnk*^*Δhepa*^ + CCl_4_ + DEN and *Jnk*^*Δhepa*^ + CCl_4_ + DEN mice treated with CM-272 as indicated in Fig. 8a. A significant transcriptional upregulation of *Phgdh* was observed in tumour bearing mice, and immunohistochemical analyses detected PHGDH in parenchymal cells and also in CCA lesions (Fig. 8b and c). Noteworthy, the antitumoral effect of CM-272 was accompanied by a marked reduction of *Phgdh* mRNA levels and PHGDH staining in regressing biliary cancer cells (Fig. 8c). The overexpression of PHGDH in *Jnk*^*Δhepa*^ + CCl_4_ + DEN mice confirmed our findings in the rat TAA model (Fig. 5b), in which we also observed upregulated G9a expression in CCA (Supplementary Fig. 7). Therefore, both CCA models reproduced the upregulation of PHGDH expression found in human iCCA (Fig. 5c). The effects of CM-272 in *Jnk*^*Δhepa*^ + CCl_4_ + DEN mice identified a pharmacological strategy to revert this pro-tumorigenic metabolic trait in vivo.

**Fig. 9:**
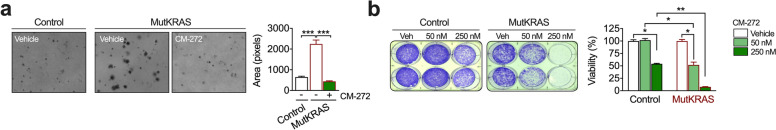
G9a targeting inhibits KRAS^G12D^ induced malignant traits in CCA cells: identification of G9a as a therapeutically relevant vulnerability in KRAS^G12D^ expressing CCA cells. **a** Anchorage-independent growth of control and KRAS^G12D^ expressing (MutKRAS) Huh28 cells treated or not with CM-272 (200 nM). Representative images of colonies formed at the end of experiments (3 weeks) and quantification of the area occupied by colonies are shown. ***p < 0.001. **b** Colony formation assay in control and MutKRAS Huh28 cells treated with CM-272 as indicated. Representative images of crystal violet-stained colonies and the corresponding quantification are shown. *p < 0.05, **p < 0.01

Since cancer cells which become dependent on KRAS-driven metabolic adaptations also become sensitive to the inhibition of these routes [46], we tested the sensitivity of control and MutKRAS Huh28 cells to the PHGDH inhibitor NCT-503 [49] and found that KRAS^G12D^ expressing cells were indeed more sensitive to this drug (GI_50_ 230 ± 5 vs 175 ± 4 μM, *p* < 0.05). Moreover, we observed that control Huh28 cells hardly grew under anchorage-independent conditions whereas MutKRAS Huh28 cells readily formed colonies in soft agar (Fig. 9a). We assessed the effect of CM-272 in this context and found a robust inhibitory activity (Fig. 9a). Next, we examined the response of control and MutKRAS Huh28 cells to CM-272 in a colony formation assay. In line with the enhanced response of MutKRAS Huh28 cells to NCT-503, we observed that KRAS^G12D^ expressing cells were significantly more sensitive to CM-272 (Fig. 9b). Interestingly, in HuCCT-1 cells, which are *KRAS* mutant, combined treatment with CM-272 and NCT-503 had a synergistic growth inhibitory effect (Supplementary Fig. 8).

**Fig. 10:**
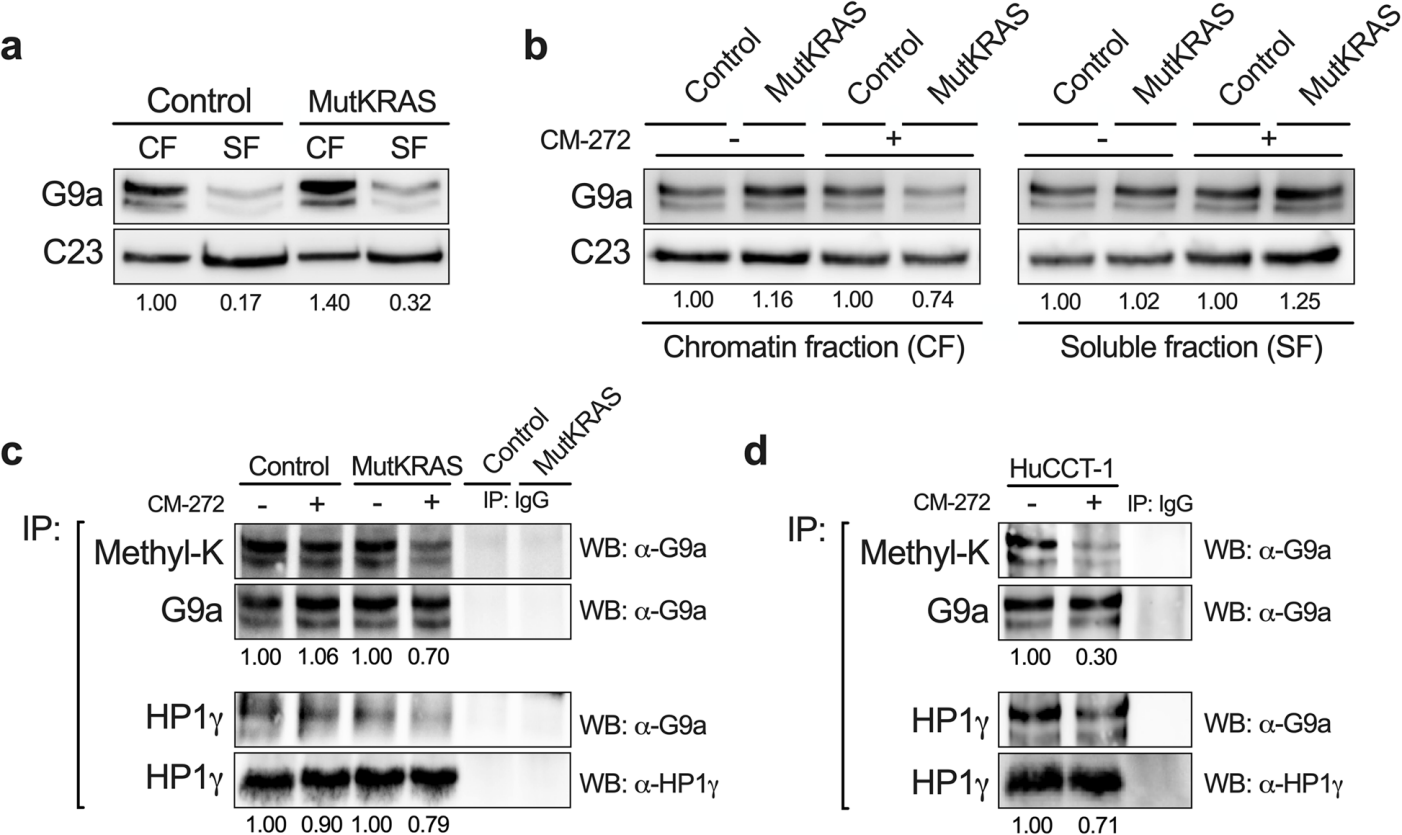
Pharmacological targeting of G9a activity in CCA cells. **a** Western blot analysis of the distribution of G9a between nuclear chromatin faction (CF) and soluble nuclear fraction (SF) in control and KRAS^G12D^ expressing (MutKRAS) Huh28 cells. Representative blots, including C23 (nucleolin) analysis as loading control, are shown. **b** Effect of G9a inhibition on the distribution of G9a between CF and SF in control and MutKRAS Huh28 cells. Cells were treated with CM-272 (200 nM) for 72 h before fractionation and western blot analyses. Representative blots, including C23 (nucleolin) analysis as loading control, are shown. **c** Effect of CM-272 on G9a methylation status and interaction with HP1γ in control and MutKRAS Huh28 cells. Cells were treated with CM-272 (200 nM) for 72 h before immunoprecipitations with an anti-G9a antibody, an anti-pan-methyllysine antibody (Methyl-K) or with an anti-HP1γ antibody, and subsequent western blot analyses to detect G9a. Corresponding immunoprecipitation controls using normal rabbit IgG are included. Representative blots are shown. **d** Effect of CM-272 on G9a methylation and interaction with HP1γ in HuCCT-1 cells. Cells were treated or not with CM272 (200 nM) for 72 h before immunoprecipitations were carried out as described in **c**. Corresponding immunoprecipitation controls using normal rabbit IgG are included. Representative blots, including levels of HP1γ in total cell lysates are shown

Together these findings indicated that in spite of having a more aggressive phenotype, MutKRAS Huh28 cells were more vulnerable to PHGDH and G9a inhibition than control Huh28 cells. In view of these results, we further explored the crosstalk between KRAS ^G12D^ and G9a in CCA cells. We previously described that cellular signaling systems involving G9a in their transcriptional responses, such as that of transforming growth factor 1β (TGF1β), promote the association of nuclear G9a with the nuclear chromatin subfraction (CF) [31]. We tested the distribution of G9a between soluble nuclear fraction (SF) and CF, observing that MutKRAS tended to have increased levels of CF bound G9a than control Huh28 cells (Fig. 10a). Most interestingly, when we evaluated the effect of CM-272 on the subnuclear distribution of G9a in both cell lines we found that G9a inhibition resulted in an enhanced dissociation of G9a from the CF in MutKRAS Huh28 cells (Fig. 10b). Besides methylating H3K9, G9a is also capable of automethylating its N-terminal domain, and this modification is functionally involved in G9a interactions with chromatin, chromatin binding proteins such as heterochromatin protein 1γ (HP1γ) and other transcriptional regulators [50]. Therefore, we evaluated G9a methylation levels in control and MutKRAS Huh28 cells under basal conditions and upon CM-272 treatment by immunoprecipitation assays using a pan-methyllysine antibody followed by G9a immunoblotting. Remarkably, we observed that G9a automethylation was significantly reduced in MutKRAS Huh28 cells while under these conditions G9a methylation status in control cells remained unaffected (Fig. 10c). Of note, CM-272 inhibition of G9a automethylation was related to a reduced association with HP1γ (Fig. 10c). The effects of CM-272 on G9a automethylation and association with HP1γ were reproduced in the iCCA cell line HuCCT-1 (Fig. 10d).

## Discussion

In this study we investigated the bile proteome and hydrophilic metabolome as a proxy of the molecular and metabolic alterations taking place in transformed biliary cells in a model of TAA-induced iCCA in rats. Consistent with observations in human iCCA, we found marked changes in bile proteins related to inflammatory pathways, including the complement and coagulation cascades and cytokine driven-pathways [51]. The biological functions of these proteins were consistent with the most relevant categories identified in our IPA analysis, and are associated with CCA development [22, 41, 45].

Among these pathways, and playing a well-recognized role in cholangiocarcinogenesis, is the cytokine IL6 [22]. IL6 levels were markedly upregulated in TAA-induced tumors, and CCA cells showed strong STAT3 activation. Within the tumor microenvironment CAFs are considered a major source of IL6, driving CCA growth in a paracrine manner [25, 51]. In addition, cancer cells can also produce IL6 [23, 52]. However, the mechanisms leading to IL6 upregulation in CCA cells are only partially known. CAFs and CCA cells engage in an extensive pro-tumorigenic crosstalk which to a great extent is mediated by the activation of EGFR signaling in CCA cells [25, 41]. We observed that the expression of EGFR ligands was also markedly upregulated in TAA-induced tumors, which also displayed significant ERK1/2 activation. In human CCA CAFs produce the EGFR ligand HB-EGF which triggers the secretion of TGFβ from CCA cells [25]. Now we demonstrate that HB-EGF can also stimulate the expression and release of IL6 from CCA cells. Moreover, in agreement with previous findings in other tumor types harboring oncogenic *KRAS* mutations [42, 43], we also found that KRAS^G12D^ can promote IL6 expression in CCA cells. Together our data suggest that the frequently observed activation of the EGFR signaling system, and/or the presence of *KRAS* mutations, may represent new mechanisms underlying the prevalent expression of IL6 in CCA tissues [23].

Other insights provided by our analysis of bile in the TAA model was the detection of ongoing oxidative stress. This prooxidant context is consistent with histological evidences of reactive oxygen species accumulation in human iCCA tissues and their procarcinogenic role. Perhaps more compelling was the detection of increased concentrations of the amino acids serine and glycine in bile from CCA bearing rats. Elevated levels of serine and glycine have been reported in a number of human cancers tissues [53], and these amino acids are considered as oncogenesis-supportive metabolites [24, 54]. Increased production of serine and glycine may result from the metabolic rewiring of glycolysis observed in cancer cells, the Warburg effect, and the activation of the serine-glycine pathway which branches off the glycolytic route. Interestingly, the majority of iCCA patients show increased tumor glucose uptake (^18^F-FDG) [45], indicating a strong dependence of these tumours on glucose metabolism, a feature also present in the rat TAA model [55]. Accordingly, in TAA-elicited tumours we found marked elevations not only in the expression of key genes driving glycolysis but also of *PHGDH* [24, 54]. Conversely, expression of the gluconeogenic gene *FBP1*, a recognized tumour suppressor gene also in CCA [56], was downregulated. We found high PHGDH expression levels in human iCCA tissues in association with more advanced disease. Interestingly, the expression of serine hydroxymethyltransferase-2 (SHMT2), a downstream enzyme in the serine-glycine pathway [24, 54], was recently reported to be overexpressed in iCCA patients with poor prognosis [57]. Different mechanisms leading to upregulation of *PHGDH* expression have been identified in cancer cells, including gene amplification, loss of *TP53* and *KRAS* mutations [24, 54]. In pancreatic cancer *KRAS* mutation reprograms glucose metabolism, upregulating the expression of serine-glycine pathway enzymes and sustaining tumor cells grow under serine starvation [46]. Our observations indicate that in CCA cells mutant *KRAS* can also drive PHGDH expression, increase glucose metabolic flux through the serine pathway, and improve survival under serine starvation. Nevertheless, other mechanisms frequently present in CCA such as c-Myc overexpression, could also participate in the activation of the serine-glycine pathway and deserve further consideration [54]. Interestingly, CCA cells expressing mutant *KRAS* were more sensitive to pharmacological PHGDH inhibition than those harboring the wild type allele, a situation that may open new therapeutic avenues for *KRAS* mutant CCAs.

Recent observations in mice with specific deletion of *G9a* in pancreatic cells evidenced the fundamental role of this epigenetic effector in *KRAS*-driven pancreatic carcinogenesis [58, 59]. In view of these compelling findings, of our previous description of the antitumor potential of G9a targeting in CCA [30], and our present observation of a direct correlation between *G9a* and *PHGDH* expression in iCCAs, we explored the crosstalk between *KRAS* and this HMT in CCA cells. Using the G9a inhibitor CM-272 we could demonstrate that G9a activity was necessary for mutant *KRAS*-mediated *PHGDH* activation. Previous reports, including ours, showed that enhanced ATF4 expression and G9a-mediated monomethylation of H3K9 at the *PHGDH* promoter could stimulate the transcription of this gene [31, 36]. While these processes are likely to be involved in the observed responses to G9a inhibition, here we decided to explore additional mechanisms and examine whether G9a nuclear distribution and methylation status could be modified in the context of *KRAS* mutation. At variance with the response to other signals (i.e. TGFβ pathway activation) [31] we did not consistently find an increased association of G9a with nuclear CF in *KRAS* mutant cells. However, inhibition of G9a activity by CM-272 resulted in enhanced dissociation of this HMT from chromatin compared with cells expressing wild type *KRAS*. As mentioned, H3K9 is methylated by G9a and this chromatin mark is recognized and bound by G9a itself leading to the recruitment of other gene regulators [50]. However, G9a can also automethylate in lysine residues within its N-terminal domain, a modification that is essential for G9a interaction with other transcriptional regulators [50]. Remarkably, we found that G9a methylation status was much more sensitive to the inhibition of G9a catalytic activity in *KRAS* mutant cells, suggesting a faster turnover of this covalent modification in cells expressing the oncogene, a mechanism that certainly deserves further exploration. Noteworthy, MutKRAS Huh28 cells were also more sensitive than control Huh28 cells to CM-272 growth inhibition. Among the transcriptional regulators interacting with methylated G9a are the HP1 proteins such as HP1γ (also known as CBX3) [60]. Moreover, a recent study demonstrated that G9a binding to HP1 proteins is dependent in part on its automethylation, and that in HP1-deficient cells G9a is released from chromatin [61]. HP1γ has been involved in both gene repression and activation [60, 62], and it is upregulated in a broad range of tumors contributing to cancer progression [63]. Interestingly, HP1γ is overexpressed in lung adenocarcinoma, and it plays a main role in *KRAS*^G12D^-driven lung tumorigenesis through its interaction with G9a-generated H3K9 methylation marks [64]. Here, we observed that inhibition of G9a automethylation reduced its interaction with HP1γ. Although the involvement of G9a-HP1γ interaction in PHGDH expression needs to be directly evaluated, it has been recently demonstrated that HP1γ can promote aerobic glycolysis in pancreatic cancer cells [62].

## Conclusions

**Fig. 11:**
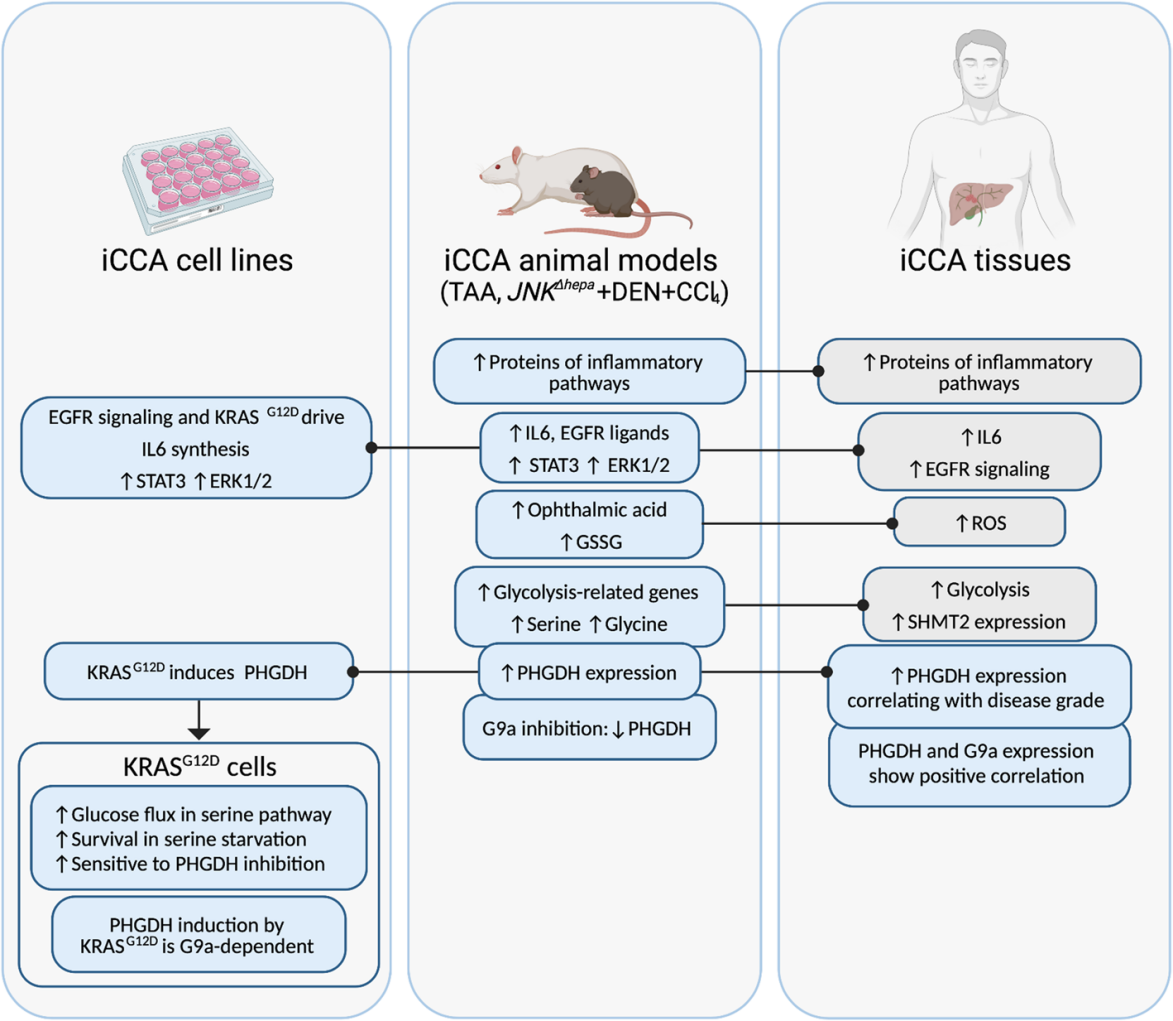
Schematic diagram of the most relevant findings in this study. Grey boxes indicate observations from other works. Created with BioRender.com

Starting from an unconventional analysis of a clinically relevant model of iCCA we have identified new processes that may be relevant to the human disease. We discovered that the EGFR signaling pathway, as well as the existence of KRAS mutations, can cause the pro-carcinogenic cytokine IL6 to be produced. Paracrine or autocrine EGFR-RAS-MAPK activation in CCA cells leading to IL6 production is a previously undiscovered mechanism in iCCA. In human iCCA, we also described that PHGDH was upregulated, and that mutant KRAS can drive PHGDH expression and glucose flux towards serine production. Functionally, *PHGDH* expression conferred a growth advantage to *KRAS*-mutant CCA cells, which also became more vulnerable to PHGDH inhibitors, a condition that may be exploited therapeutically. Finally, we provided pharmacological evidence that G9a is a component of KRAS-mediated glucose metabolism via the serine-glycine pathway. Most of these data were also depicted in our rat TAA model, as they were for many other histological, cellular, and molecular features. However, due to the size of the animals, conducting pharmacological experiments is difficult. The inclusion of a mouse model of human CCA pathogenesis in our study confirmed that PHGDH expression expression is activated in CCA and that G9a inhibition has therapeutic potential. These findings suggest that interference with G9a activity could be particularly useful for the treatment of KRAS mutant CCAs, further supporting the potential of epigenetic therapies for this devastating disease. The most relevant findings of this study are summarized in Fig. 11.

## Supplementary Information

**Additional file 1.**

**Additional file 2: Supplementary Figure 1.** Rat TAA model of CCA. a. Body weight and liver index at different time-points in control (Vehicle) and TAA treated rats. *p < 0.05. b. Liver-related serum parameters at different time-points in control (Vehicle) and TAA treated rats. *p < 0.05. c. Representative photographs of livers from control (Vehicle) and TAA treated rats at 30 weeks of treatment. Numerous tumoural lesions are visible in the surface of TAA treated rats. d. Representative images of H&E and Sirius Red staining, as well as immunohistochemical detection of CK-19, in liver tissue sections from control (Vehicle) and TAA treated rats, peritumoural and tumoural tissues, at 30 weeks of treatmentok. Created with BioRender.com.

**Additional file 3: Supplementary Figure 2.** Validation of the expression of selected genes corresponding to proteins elevated in the bile of TAA treated rats. a. qRT-PCR analysis of the expression of the indicated genes in liver tissue samples from control rats (Vehicle), peritumoural liver tissues and tumoural tissues. *p < 0.05, **p < 0.01, ***p < 0.001. b. Representative images of the immunohistochemical analysis of SPP1, YWHAQ and S100A11 proteins in liver tissue sections from from control rats (Vehicle), peritumoural liver tissues and tumoural tissues.

**Additional file 4: Supplementary Figure 3.** qRT-PCR analysis of the expression of the indicated genes in liver tissue samples from control rats (Vehicle), peritumoural liver tissues and tumoural tissues. *p < 0.05, **p < 0.01, ***p < 0.001.

**Additional file 5: Supplementary Figure 4.** *PHGDH* expression knockdown and CCA cells growth. a. HuCCT-1 and TFK-1 cells were transfected with *PHGDH* specific siRNAs (siPHGDH) or control siRNA (siGL) and 48 h later PHGDH protein levels were analyzed by western blotting. Representative blots, including HSP90 and β-ACTIN analyses as loading controls are shown. b. Growth of HuCCT-1 and TFK-1 cells transfected with siPHGDH or control siGL siRNAs. **p < 0.01, ***p < 0.001.

**Additional file 6: Supplementary Figure 5.** Effect of CM-272 on the in vitro growth of organoids established from a core biopsy obtained from patient with iCCA. Organoids were treated for 10 days with the indicated concentrations of CM-272, or the equivalent volume of vehicle at maximal CM-272 concentration (< 0.1% DMSO in culture medium).

**Additional file 7: Supplementary Figure 6.** Correlation between the expression of *G9a* and that of *PHGDH* in iCCA tumor tissues (*n* = 122) from the EGAD00001001693 dataset.

**Additional file 8: Supplementary Figure 7.** Immunohistochemical analysis of G9a in liver tissue samples from control rats (Vehicle), peritumoural liver tissues and tumoural tissues. Representative images are shown.

**Additional file 9: Supplementary Figure 8.** Combination study of the growth inhibitory effects of CM-272 and NCT-503 in HuCCT-1 cells. Grey bars denote the existence of synergism (combination index, CI < 1) at the indicated doses.

**Additional file 10.**

## Data Availability

The mass spectrometry proteomics data have been deposited to the ProteomeXchange Consortium via the PRIDE partner repository with the dataset identifier PXD032802. Sequences of primers used in this study are available upon request.

## Abbreviations

ACN: Acetonitrile
Aldo A: Aldolase A
ALP: Alkaline Phosphatase
ALT: Alanine Aminotransferase
ANXA2: Annexin A2
Areg: Amphiregulin
AST: Aspartate Aminotransferase
ATF4: Activating Transcription Factor 4
BA: Bile Acid
CAF: Cancer Associated Fibroblasts
CCA: Cholangiocarcinoma
CD44: Cluster of Differentiation 44
CE-MS: Capillary Electrophoresis-Mass Spectrometry
CF: Chromatin Subfraction
cfDNA: Cell Free DNA
CK19: Cytokeratin 19
ctDNA: Circulating Tumor DNA
DEN: Diethylnitrosamine
DMBT1: Deleted in Malignant Brain Tumors 1
eCCA: Extrahepatic Cholangiocarcinoma
EGFR: Epidermal Growth Factor Receptor
ERCP: Endoscopic Retrograde Cholangiopancreatography
Ereg: Epiregulin
ESI: Electrospray Ionization Source
Fbp1: Fructose-1,6-BisPhosphatase 1
Fcgbp: Fc Gamma Binding Protein
FFPE: Formalin-Fixed and Paraffin Embedded
GI_50_: Growth Inhibitory 50
GSSG: Gluthathione Disulfide
H&E: Hematoxylin and Eosin
Hb-egf: Heparin-Binding Epidermal Growth Factor
Hk-I: Hexokinase 1
HMT: Histone Methyltransferase
HP1γ: Heterochromatin Protein 1 gamma
iCCA: Intrahepatic Cholangiocarcinoma
IL6: Interleukin 6
IPA: Ingenuity Pathway Analysis
Jnk 1/2: c-Jun Kinase 1/2
Jnk^Δhepa^: hepatocellular Jnk 1/2 deletion
LCN2: Lipocalin 2
LGALS3BP: L-Galectin-3-Binding Protein
MAPK: Mitogen Activated Protein Kinase
MEK: Mitogen-activated protein kinase kinase
MSLN: Mesothelin
PCA: Principal Component Analysis
Pfkfb3: 6-PhosphoFructo-2-Kinase/Fructose-2,6-Bisphosphatase 3
Phgdh: Phosphoglycerate Dehydrogenase
PI3K: Phosphatidylinositol 3 kinase
qRT-PCR: Quantitative Real Time PCR
SF: Soluble Nuclear Fraction
SFN: Stratifin
SHMT2: Serine-hydroxymethyltransferase 2
SPP1: Osteopontin
STAT3: Signal Transducer and Activator of Transcription 3
TAA: Thioacetamide
TGF1β: Transforming Growth Factor 1 beta
UPLC-ToF-MS: Ultra Performance Liquid Chromatography-Time-of-Flight mass spectrometry
VIM: Vimentin
YWHAQ: 14-3-3 theta
YWHAS: 14-3-3 sigma
