# Supplementary material for: New molecular mechanisms in cholangiocarcinoma: signals triggering interleukin-6 production in tumor cells and KRAS co-opted epigenetic mediators driving metabolic reprogramming
Source: J Exp Clin Cancer Res. 2022 May 26;41:183. doi: 10.1186/s13046-022-02386-2 (PMC9134609; doi:10.1186/s13046-022-02386-2)
Supplement: Supplementary file 1 — Additional file 1. [file 13046_2022_2386_MOESM1_ESM.docx]

***Additional materials and methods***

*Analysis of bacterial DNA in rat liver tissues*

Total DNA was isolated from 50 mg of rat liver tissues. After homogenization in 100 μL 10mM Tris-1 mM EDTA, pH 8.0 (TE) buffer DNA was purified using the Maxwell RSC DNA extraction kit (#AS1620, Promega, Madison, WI, USA) with the Promega Maxwell RSC Instrument. DNA concentrations were quantified with a Nanodrop instrument and 100 ng of total DNA were used for quantitative real-time PCR (qRT-PCR). Bacterial 16S rDNA was amplified as described before (1) and data were normalized by qRT-PCR analysis of rat 18S rDNA contents.

*Serum biochemistry and bile acids (BA) determinations*

Serum levels of alanine aminotransferase (ALT), aspartate aminotransferase (AST), alkaline phosphatase (ALP), bilirubin and BA were measured as previously reported (2).

*Bile metabolites analysis*

Fifty μl of bile was mixed with 150 μl of cold acetonitrile (ACN) (-20°C), after vortexing, samples were centrifuged (10 min at 4°C, 2000 *xg*) and supernatants were evaporated to dryness and reconstituted in 50 μl of 0.1 M formic acid containing 0.2 mM methionine sulfone as internal standard. Bile untargeted capillary electrophoresis-mass spectrometry (CE−MS) analyses were performed using a CE 7100 (Agilent Technologies, Wilmington, USA) coupled to an Accurate-Mass TOF−MS system 6224 (Agilent Technologies). The coupling was equipped with an electrospray ionization source (ESI) (3). The analysis was performed using a modified method for a global profiling of the sample as we recently described (4). After the untargeted screening, glycine, L-serine, L-ornithine, L-lysine, L-arginine, DL-citrulline, L-cystine, ophthalmic acid, and glutathione disulfide (GSSG) were selected and quantitated as described (5).

*Gene expression analyses*

RNA was extracted from cells and tissues as described (6). Total RNA was extracted using the automated Maxwell system (Promega). Quantitative reverse transcription PCR (qRT-PCR) was performed as reported, and gene expression was normalized relative to that of the housekeeping gene *H3F3A* also as described (6). Primers sequences are available upon request. Fastq files of RNAseq from 122 samples of iCCA (7) were downloaded from the European Genome-phenome Archive (https://www.ebi.ac.uk/ega/datasets/EGAD00001001693) and correlation between *PHGDH* and *G9a* gene expression was analyzed as described (6).

*Immunoblots and immunoprecipitation assays*

Cells were lysed in RIPA buffer and homogenates were subjected to immunoblot (Western blot) analysis as reported (8). Immunoprecipitation analyses were performed as described (9). Antibodies used are reported in Supplementary Table 1. For quantification of immunoblot signals, indicated in figures, we used the Image Lab Software from Bio-Rad Laboratories.

*Sample processing and serine UPLC-ToF-MS analysis*

Cells were lysed in ice-cold methanol/water (50/50 %v/v) for 15 min, three plates were pooled per point (cell type and treatment), and each point was measured in triplicate. Homogenates were extracted with the same volume of chloroform (1 h, 4°C), centrifuged (30 min, 13 rpm, 4°C) and the aqueous phase was kept at -80°C for 20 min. Subsequently, samples were evaporated (speedvac) and pellets were resuspended in water/acetonitrile (MeCN)(40/60 % v/v). Concentrations of metabolites were determined with a semi-quantitative method. Calibration curves were obtained by measuring serial dilutions of a pooled standard mixture in resuspension solution. The concentrations in the dilutions ranged from 100 µM to 0.025 µM. Samples were measured with a UPLC system (Acquity, Waters Inc.) coupled to a Time-of-Flight mass spectrometer (ToF-MS, SYNAPT G2, Waters Inc.). A 2.1 x 100 mm, 1.7 µm BEH amide column (Waters Inc.), thermostated at 40 °C, was used to separate the analytes before entering the MS. Solvent A (aqueous phase) consisted of 99.5% water, 0.5% formic acid and 20 mM ammonium formate while solvent B (organic phase) consisted of 29.5% water, 70% acetonitrile, 0.5% formic acid and 1 mM ammonium formate. To obtain a good separation of analytes the following gradient was used: from 5% A to 50% A in 2.4 minutes in curved gradient (#8, as defined by Waters Inc.), from 50% A to 99.9% A in 0.2 minutes constant at 99.9% A for 1.2 minutes, back to 5% A in 0.2 minutes. The flow rate was 0.250 mL/min and the injection volume was 4 µL. All samples were injected randomly. After every 9 injections a QC sample was injected. The MS was operated in positive electrospray ionization in full scan mode. The cone voltage was 25 V and capillary voltage was 250 V. Source temperature was set to 120 °C and capillary temperature to 450 °C. The flow of the cone and desolvation gas (both nitrogen) were set to 5 L/h and 600 L/h, respectively. A 2 ng/mL leucine-enkephalin solution in water/acetonitrile/formic acid (49.9/50/0.1 %v/v/v) was infused at 10 µL/min and used for a lock mass which was measured each 36 seconds for 0.5 seconds. Spectral peaks were automatically corrected for deviations in the lock mass. Extracted ion traces for relevant analytes were obtained in a 20 mDa window in their expected m/z-channels. These traces were subsequently smoothed and peak areas integrated with TargetLynx software. Signals of labelled analytes were corrected for naturally occurring isotopes. These calculated raw signals were adjusted for by median fold-change (MFC) adjustment. The MFC is based on the total amount of detected mass spectrometric features (unique retention time/mass pairs). The calculations and performance of the MFC adjustment factors were performed as described (10,11).

1. Nadkarni MA, Martin FE, Jacques NA, Hunter N. Determination of bacterial load by real-time PCR using a broad-range (universal) probe and primers set. Microbiology [Internet]. 2002 [cited 2022 Feb 28];148(Pt 1):257–66. Available from: https://pubmed.ncbi.nlm.nih.gov/11782518/

2. Alvarez-Sola G, Uriarte I, Latasa MU, Fernandez-Barrena MG, Urtasun R, Elizalde M, et al. Fibroblast growth factor 15/19 (FGF15/19) protects from diet-induced hepatic steatosis: development of an FGF19-based chimeric molecule to promote fatty liver regeneration. Gut. 2017;66(10):1818–28.

3. Albóniga OE, Jiménez D, Sánchez-Conde M, Vizcarra P, Ron R, Herrera S, et al. Metabolic Snapshot of Plasma Samples Reveals New Pathways Implicated in SARS-CoV-2 Pathogenesis. J Proteome Res [Internet]. 2022 [cited 2022 Feb 17];acs.jproteome.1c00786. Available from: https://pubmed.ncbi.nlm.nih.gov/35133846/

4. Gil-De-La-Fuente A, Godzien J, Saugar S, Garcia-Carmona R, Badran H, Wishart DS, et al. CEU Mass Mediator 3.0: A Metabolite Annotation Tool. J Proteome Res [Internet]. 2019 Feb 1 [cited 2022 Feb 17];18(2):797–802. Available from: https://pubmed.ncbi.nlm.nih.gov/30574788/

5. Naz S, Garcia A, Rusak M, Barbas C. Method development and validation for rat serum fingerprinting with CE-MS: application to ventilator-induced-lung-injury study. Anal Bioanal Chem [Internet]. 2013 May 1 [cited 2022 Feb 17];405(14):4849–58. Available from: https://pubmed.ncbi.nlm.nih.gov/23535741/

6. Colyn L, Bárcena-Varela M, Álvarez-Sola G, Latasa MU, Uriarte I, Santamaría E, et al. Dual Targeting of G9a and DNA Methyltransferase-1 for the Treatment of Experimental Cholangiocarcinoma. Hepatology [Internet]. 2021 Jun 1 [cited 2021 Dec 7];73(6):2380–96. Available from: https://pubmed.ncbi.nlm.nih.gov/33222246/

7. Nakamura H, Arai Y, Totoki Y, Shirota T, Elzawahry A, Kato M, et al. Genomic spectra of biliary tract cancer. Nat Genet [Internet]. 2015 Sep 10 [cited 2019 Apr 16];47(9):1003–10. Available from: http://www.nature.com/articles/ng.3375

8. Bárcena-Varela M, Caruso S, Llerena S, Álvarez-Sola G, Uriarte I, Latasa MU, et al. Dual Targeting of Histone Methyltransferase G9a and DNA-Methyltransferase 1 for the Treatment of Experimental Hepatocellular Carcinoma. Hepatology [Internet]. 2019 Feb [cited 2019 Apr 16];69(2):587–603. Available from: http://doi.wiley.com/10.1002/hep.30168

9. Recalde M, Gárate-Rascón M, Elizalde M, Azkona M, Latasa MU, Bárcena-Varela M, et al. The splicing regulator SLU7 is required to preserve DNMT1 protein stability and DNA methylation. Nucleic Acids Res [Internet]. 2021 Sep 7 [cited 2022 Feb 16];49(15):8592–609. Available from: https://pubmed.ncbi.nlm.nih.gov/34331453/

10. Dieterle F, Ross A, Schlotterbeck G, Senn H. Probabilistic quotient normalization as robust method to account for dilution of complex biological mixtures. Application in1H NMR metabonomics. Anal Chem. 2006 Jul 1;78(13):4281–90.

11. Veselkov KA, Vingara LK, Masson P, Robinette SL, Want E, Li J V., et al. Optimized preprocessing of ultra-performance liquid chromatography/mass spectrometry urinary metabolic profiles for improved information recovery. Anal Chem [Internet]. 2011 Aug 1 [cited 2022 Mar 10];83(15):5864–72. Available from: https://pubmed.ncbi.nlm.nih.gov/21526840/
