# Supplementary figures and images for: New molecular mechanisms in cholangiocarcinoma: signals triggering interleukin-6 production in tumor cells and KRAS co-opted epigenetic mediators driving metabolic reprogramming
Source: J Exp Clin Cancer Res. 2022 May 26;41:183. doi: 10.1186/s13046-022-02386-2 (PMC9134609; doi:10.1186/s13046-022-02386-2)

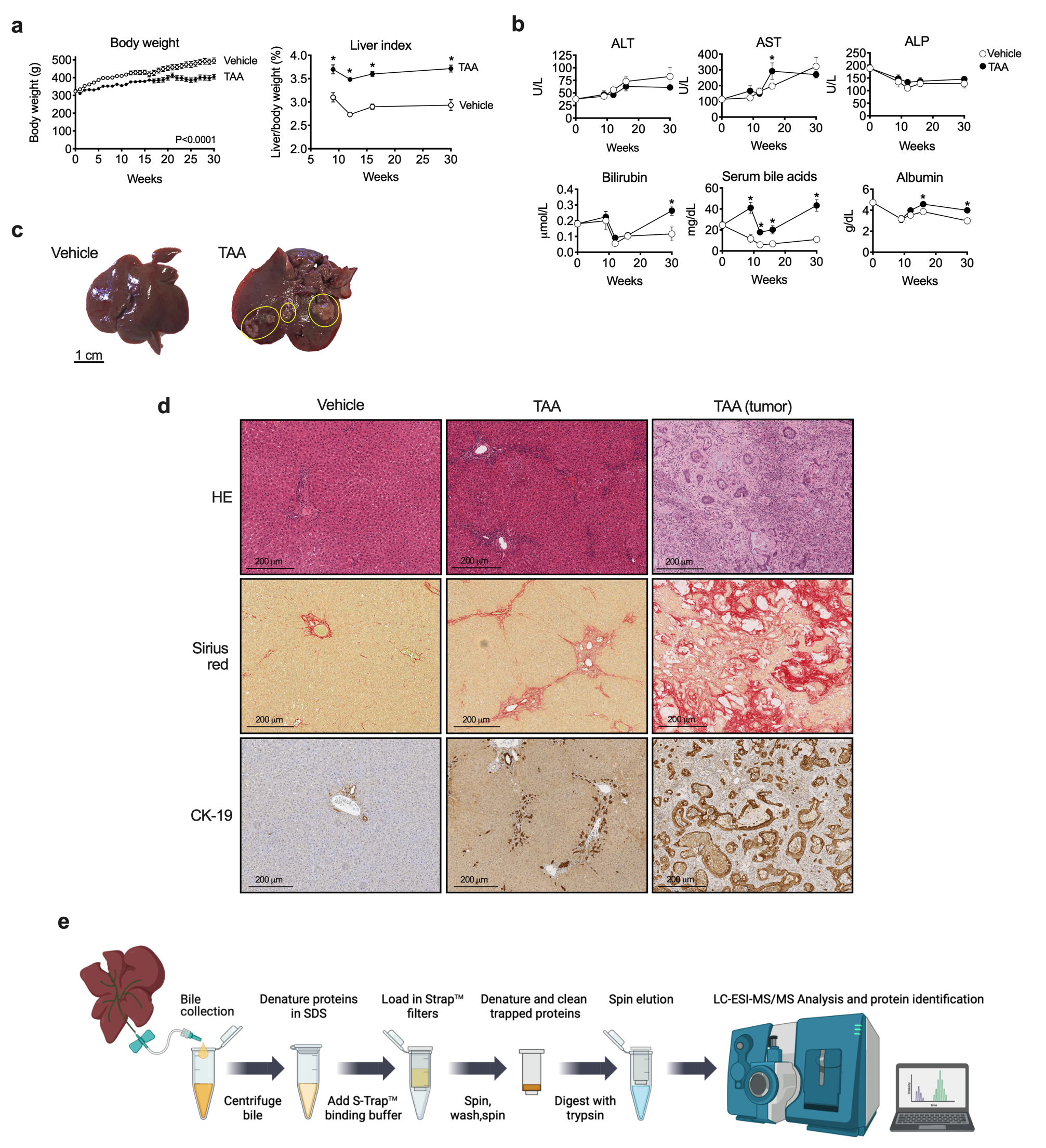

Supplement: Supplementary file 2 — Additional file 2: Supplementary Figure 1. Rat TAA model of CCA. a. Body weight and liver index at different time-points in control (Vehicle) and TAA treated rats. *p < 0.05. b. Liver-related serum parameters at different time-points in control (Vehicle) and TAA treated rats. *p < 0.05. c. Representative photographs of livers from control (Vehicle) and TAA treated rats at 30 weeks of treatment. Numerous tumoural lesions are visible in the surface of TAA treated rats. d. Representative images of H&E and Sirius Red staining, as well as immunohistochemical detection of CK-19, in liver tissue sections from control (Vehicle) and TAA treated rats, peritumoural and tumoural tissues, at 30 weeks of treatmentok. Created with BioRender.com. [file 13046_2022_2386_MOESM2_ESM.tiff]

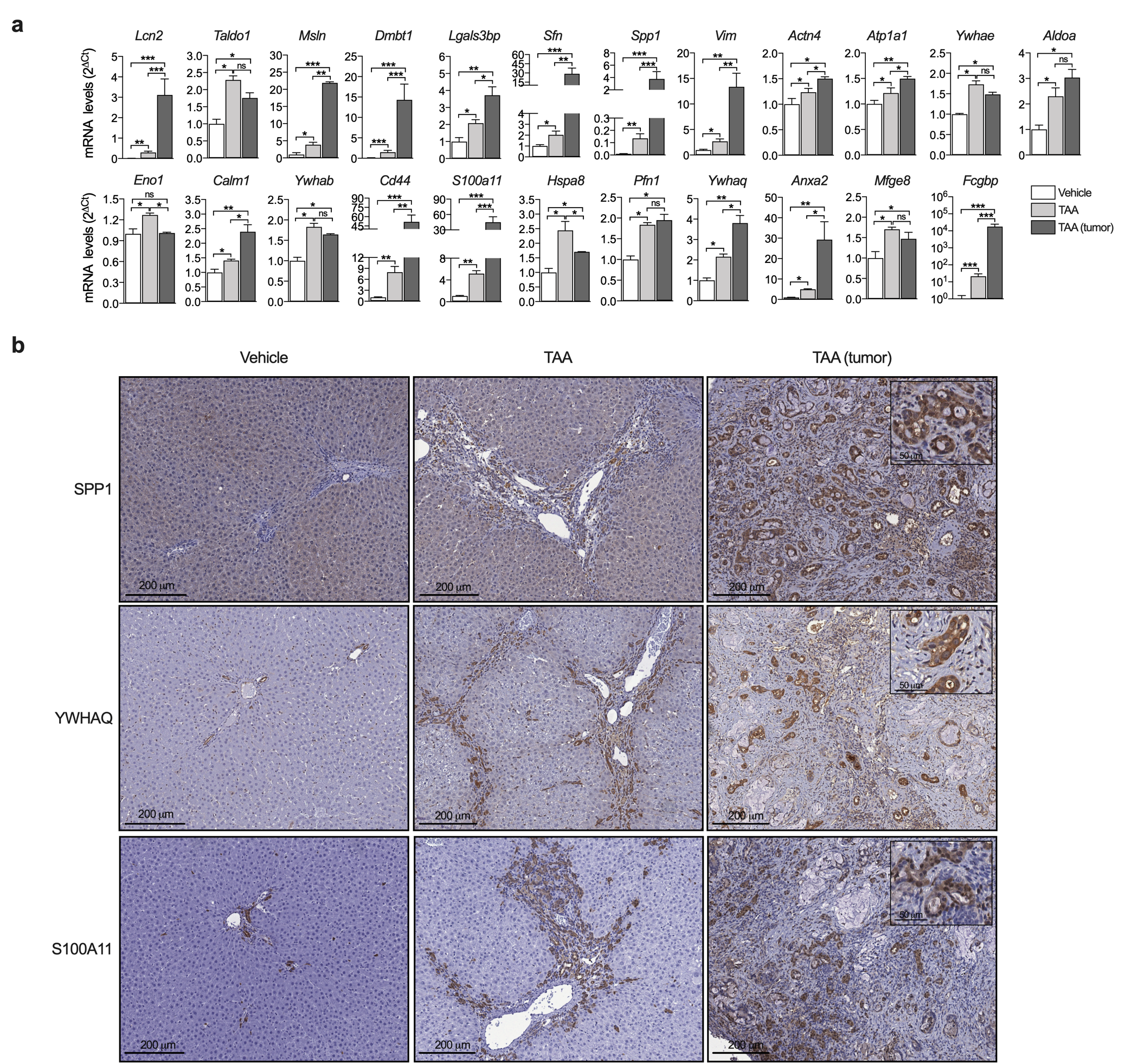

Supplement: Supplementary file 3 — Additional file 3: Supplementary Figure 2. Validation of the expression of selected genes corresponding to proteins elevated in the bile of TAA treated rats. a. qRT-PCR analysis of the expression of the indicated genes in liver tissue samples from control rats (Vehicle), peritumoural liver tissues and tumoural tissues. *p < 0.05, **p < 0.01, ***p < 0.001. b. Representative images of the immunohistochemical analysis of SPP1, YWHAQ and S100A11 proteins in liver tissue sections from from control rats (Vehicle), peritumoural liver tissues and tumoural tissues. [file 13046_2022_2386_MOESM3_ESM.tiff]

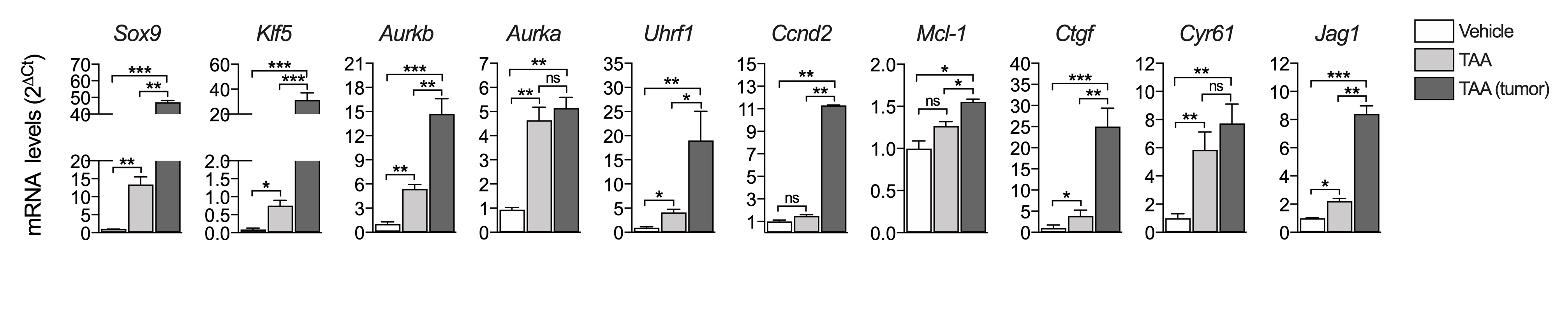

Supplement: Supplementary file 4 — Additional file 4: Supplementary Figure 3. qRT-PCR analysis of the expression of the indicated genes in liver tissue samples from control rats (Vehicle), peritumoural liver tissues and tumoural tissues. *p < 0.05, **p < 0.01, ***p < 0.001. [file 13046_2022_2386_MOESM4_ESM.tiff]

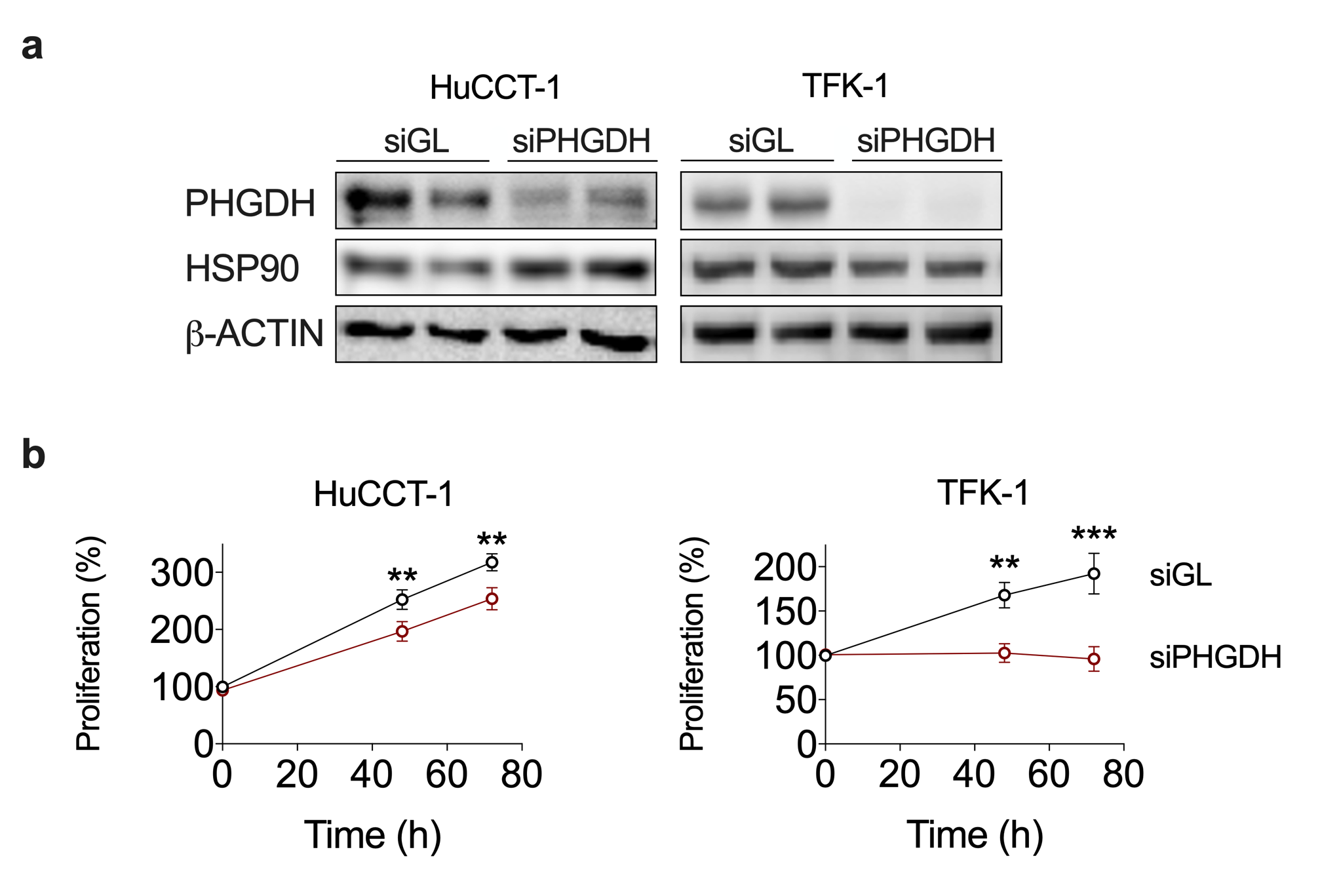

Supplement: Supplementary file 5 — Additional file 5: Supplementary Figure 4. PHGDH expression knockdown and CCA cells growth. a. HuCCT-1 and TFK-1 cells were transfected with PHGDH specific siRNAs (siPHGDH) or control siRNA (siGL) and 48 h later PHGDH protein levels were analyzed by western blotting. Representative blots, including HSP90 and β-ACTIN analyses as loading controls are shown. b. Growth of HuCCT-1 and TFK-1 cells transfected with siPHGDH or control siGL siRNAs. **p < 0.01, ***p < 0.001. [file 13046_2022_2386_MOESM5_ESM.tiff]

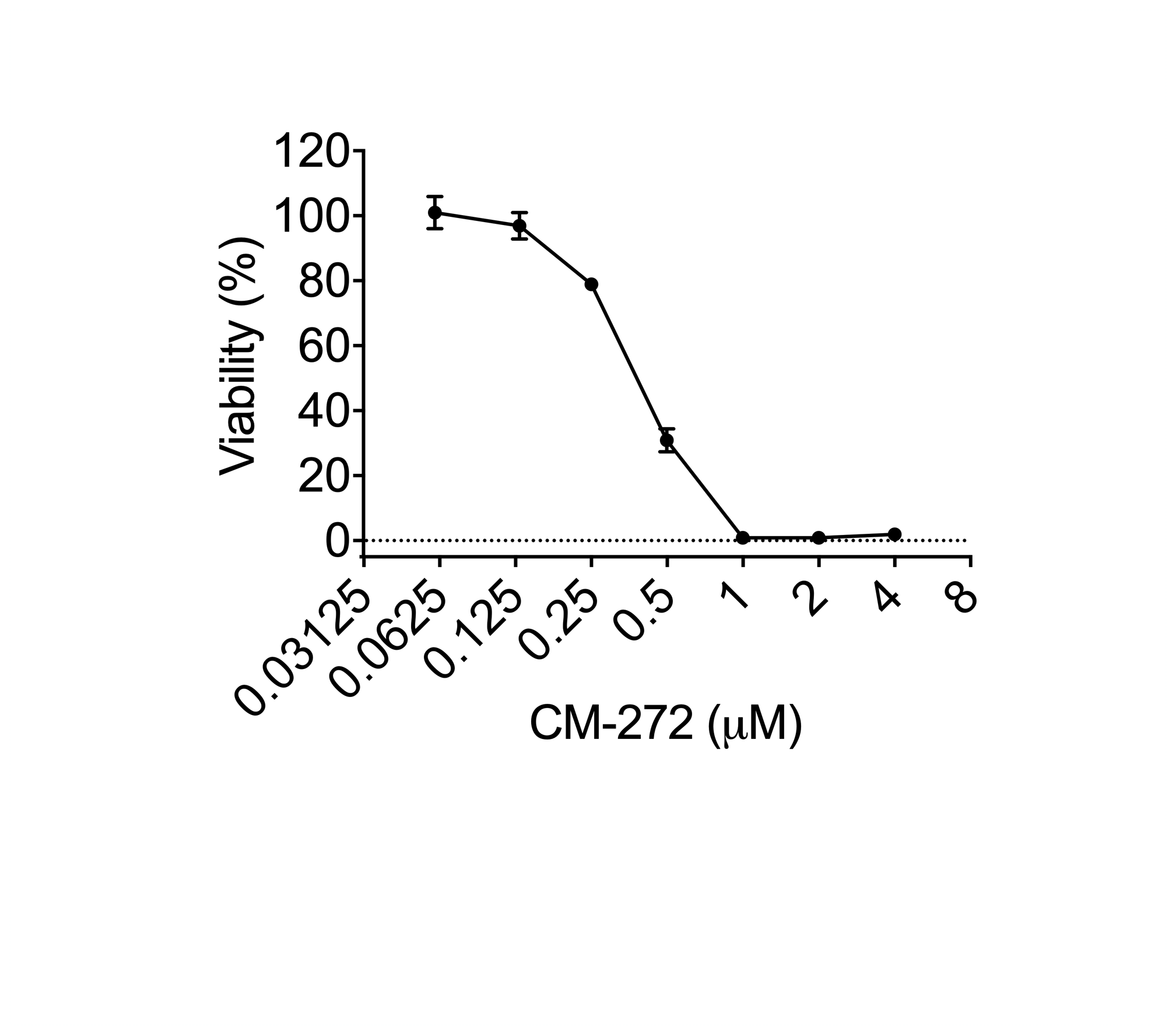

Supplement: Supplementary file 6 — Additional file 6: Supplementary Figure 5. Effect of CM-272 on the in vitro growth of organoids established from a core biopsy obtained from patient with iCCA. Organoids were treated for 10 days with the indicated concentrations of CM-272, or the equivalent volume of vehicle at maximal CM-272 concentration (< 0.1% DMSO in culture medium). [file 13046_2022_2386_MOESM6_ESM.tiff]

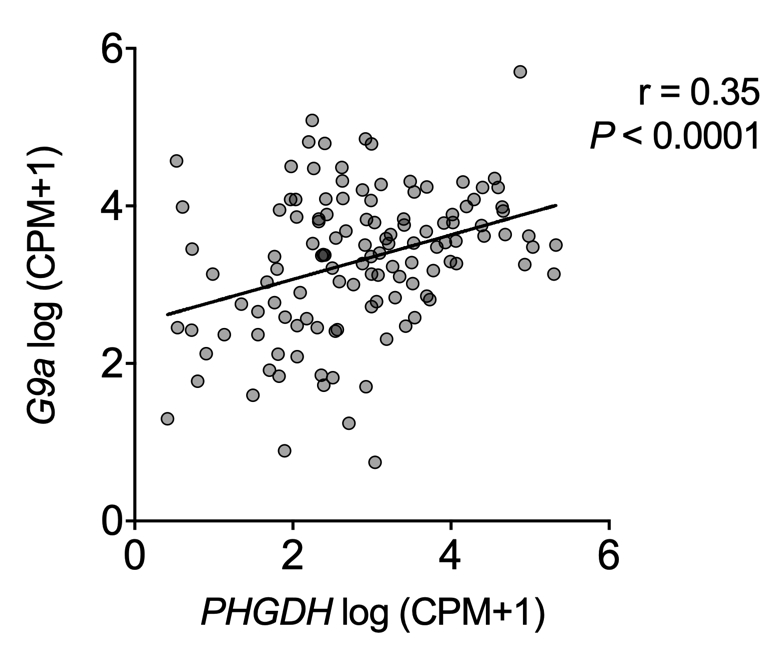

Supplement: Supplementary file 7 — Additional file 7: Supplementary Figure 6. Correlation between the expression of G9a and that of PHGDH in iCCA tumor tissues (n = 122) from the EGAD00001001693 dataset. [file 13046_2022_2386_MOESM7_ESM.tiff]

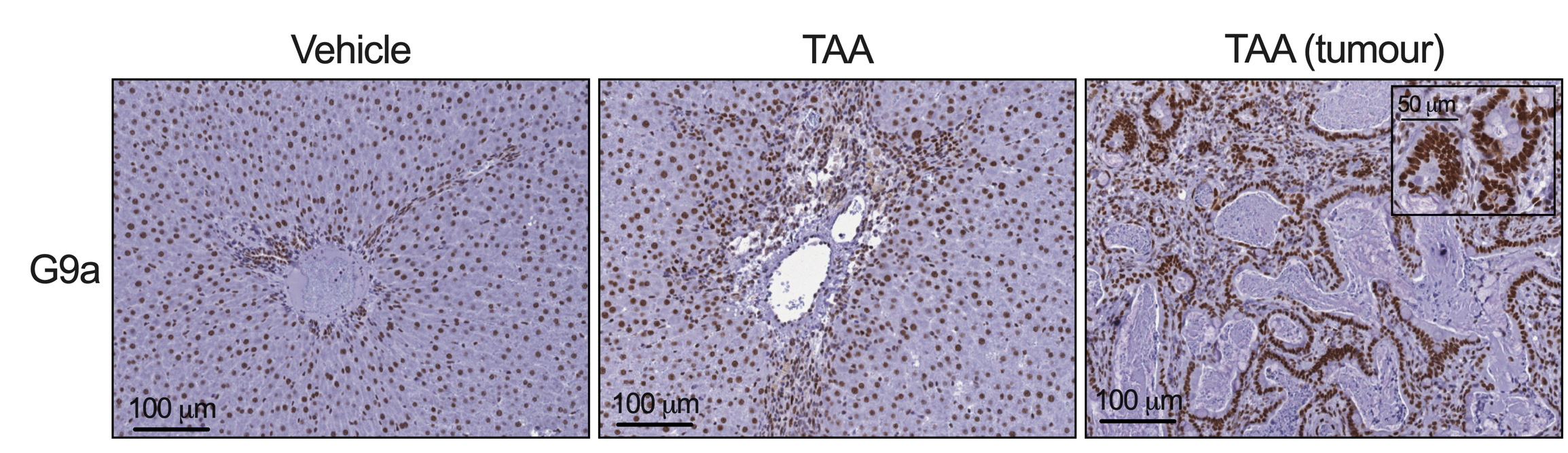

Supplement: Supplementary file 8 — Additional file 8: Supplementary Figure 7. Immunohistochemical analysis of G9a in liver tissue samples from control rats (Vehicle), peritumoural liver tissues and tumoural tissues. Representative images are shown. [file 13046_2022_2386_MOESM8_ESM.tiff]

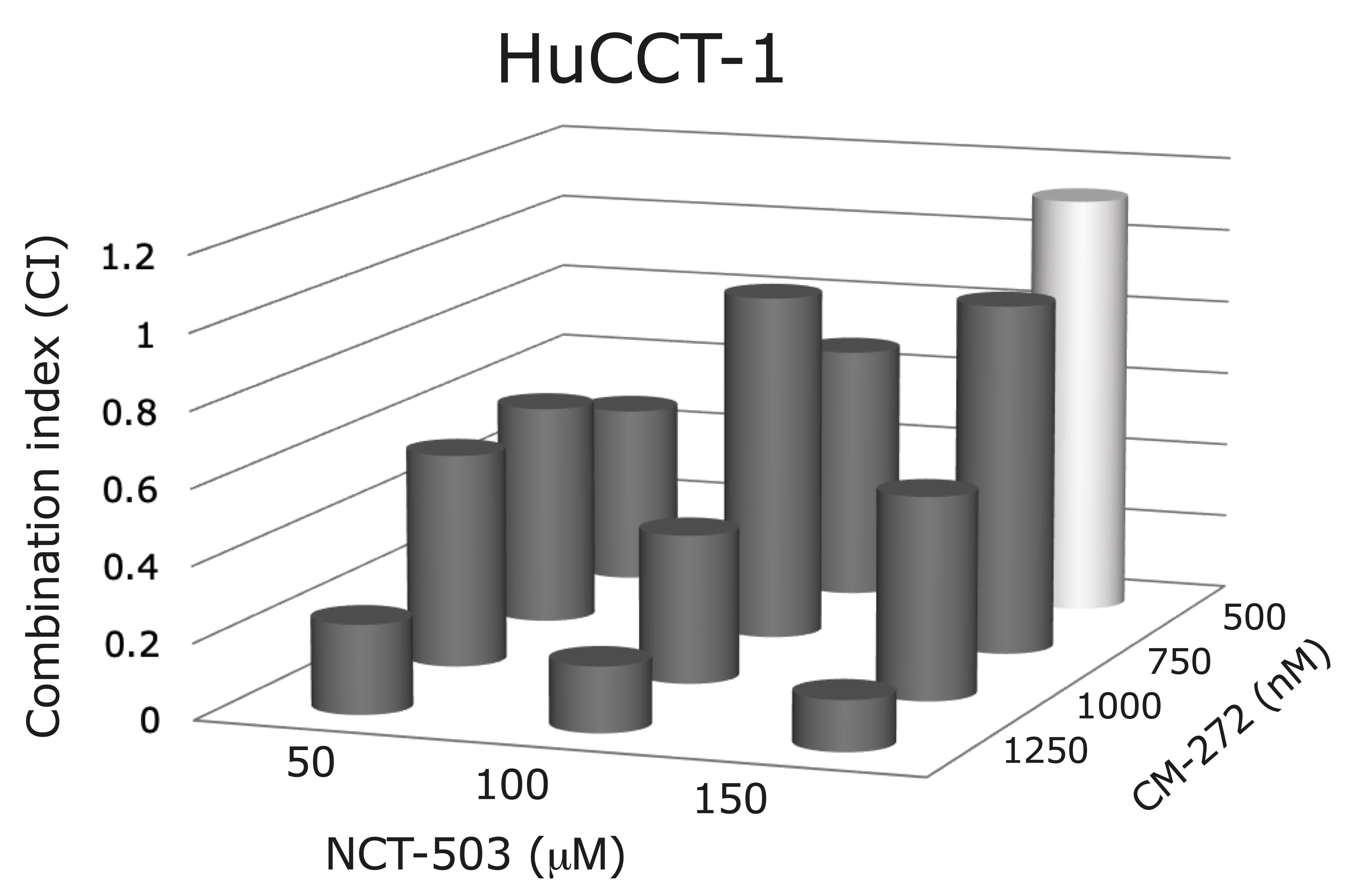

Supplement: Supplementary file 9 — Additional file 9: Supplementary Figure 8. Combination study of the growth inhibitory effects of CM-272 and NCT-503 in HuCCT-1 cells. Grey bars denote the existence of synergism (combination index, CI < 1) at the indicated doses. [file 13046_2022_2386_MOESM9_ESM.tiff]
