# Supplementary material for: New molecular mechanisms in cholangiocarcinoma: signals triggering interleukin-6 production in tumor cells and KRAS co-opted epigenetic mediators driving metabolic reprogramming
Source: J Exp Clin Cancer Res. 2022 May 26;41:183. doi: 10.1186/s13046-022-02386-2 (PMC9134609; doi:10.1186/s13046-022-02386-2)
Supplement: Supplementary file 10 — Additional file 10. [file 13046_2022_2386_MOESM10_ESM.docx]

**Supplementary Table 1**

| **List of antibodies** | |  | |  | |  |  |  |
| --- | --- | --- | --- | --- | --- | --- | --- | --- |
|  | |  | |  | |  |  | |
| **Antibody** | **Application** | | **Supplier** | **Reference** | **Dilution** | |  |  |
| β-Actin | WB | | Abcam | ab6276 | 1:25000 | |  |  |
| C23 (nucleolin) | WB | | Santa Cruz | sc-8031 | 1:1000 | |  |  |
| pEGFR (Tyr1148) | WB | | Cell Signaling | 4044 | 1:1000 | |  |  |
| EGFR | WB | | Millipore | 06-847 | 1:1000 | |  |  |
| FBP1 | WB | | Sigma-Aldrich | HPA005857 | 1:1000 | |  |  |
| G9a | WB | | Abcam | ab185050 | 1:1000 | |  |  |
| GAPDH | WB | | CellSignaling | 2118 | 1:5000 | |  |  |
| H3 | WB | | Millipore | 05-928 | 1:1000 | |  |  |
| HSP90 | WB | | Cell Signaling | 4874 | 1:1000 | |  |  |
| KRAS G12D | WB | | Cell Signaling | 14429 | 1:1000 | |  |  |
| pMEK1/2 (Ser217/221) | WB | | Cell Signaling | 9121 | 1:1000 | |  |  |
| MEK | WB | | Cell Signaling | 9122 | 1:1000 | |  |  |
| Phospho-p44/42 MAPK (Thr202/Tyr204) | WB | | Cell Signaling | 4376 | 1:1000 | |  |  |
| p44/42 MAPK (Erk1/2) | WB | | Cell Signaling | 9102 | 1:1000 | |  |  |
| PHGDH | WB | | Abcam | ab240744 | 1:500 | |  |  |
| p-STAT3 (Tyr705) | WB | | Cell Signaling | 9131S | 1:1000 | |  |  |
| STAT3 | WB | | Upstate | 06-596 | 1:1000 | |  |  |
| CK19 | IHC | | Cell Applications | CP10405 | 1:200 | |  |  |
| G9a | IHC | | Abcam | ab185050 | 1:3000 | |  |  |
| Phospho-p44/42 MAPK (Thr202/Tyr204) | IHC | | Cell Signaling | 4376 | 1:1000 | |  |  |
| PHGDH | IHC | | Abcam | ab125865 | 1:3000 | |  |  |
| p-STAT3 (Tyr705) | IHC | | Santa Cruz | sc-8059 | 1:500 | |  |  |
| S100A11 | IHC | | Abcam | ab180735 | 1:2000 | |  |  |
| SPP1 (Osteopontin) | IHC | | Abcam | ab63856 | 1:200 | |  |  |
| YWHAQ | IHC | | Abcam | ab183075 | 1:200 | |  |  |
| HP1γ/CBX3 | IP/WB | | Abcam | ab217999 | 5μg for 500 μg of protein | |  |  |
| G9a | IP/WB | | Sigma-Aldrich | G6919 | 5μg for 500 μg of protein | |  |  |
| IgG | IP | | Cell Signaling | 2729 | 5μg for 500 μg of protein | |  |  |
| Pan-methyllysine | IP | | Abcam | ab23366 | 5μg for 500 μg of protein | |  |  |
| Anti-rabbit IgG peroxidase | Secondary for WB | | Sigma-Aldrich | A0545 | 1:5000 | |  |  |
| Anti-mouse IgG peroxidase | Secondary for WB | | Sigma-Aldrich | A0168 | 1:5000 | |  |  |
| EnVision + Dual Link System-HRP (DAB+) | Secondary for IHQ | | Dako | K4065 | Not diluted | |  |  |
